# Glutathione hybrid poly (beta-amino ester)-plasmid nanoparticles for enhancing gene delivery and biosafety

**DOI:** 10.1016/j.jare.2024.07.038

**Published:** 2024-08-02

**Authors:** Songwei Tan, Caiyan Yuan, Yuhe Zhu, Shuangyan Chang, Qianru Li, Jiahui Ding, Xueqin Gao, Rui Tian, Zhiqiang Han, Zheng Hu

**Affiliations:** a School of Pharmacy, Tongji Medical College, Huazhong University of Science and Technology, Wuhan 430030, China; b The First Hospital of Nanchang, Nanchang 330008, China; c Generulor Co., Ltd., Zhuhai 519000, Guangdong, China; d Department of Pharmacy, Union Hospital, Tongji Medical College, Huazhong University of Science and Technology, Wuhan 430022, China; e Department of Obstetrics and Gynecology, Tongji Hospital, Tongji Medical College, Huazhong University of Science and Technology, Wuhan 430030, China; f Department of Obstetrics and Gynecology, Shanxi Bethune Hospital, Taiyuan 030032, China; g Department of Gynecologic Oncology, Women and Children’s Hospital Affiliated to Zhongnan Hospital of Wuhan University, Wuhan 430071, Hubei, China; h Department of Radiation and Medical Oncology, Zhongnan Hospital of Wuhan University, Wuhan 430071, Hubei, China; i Hubei Key Laboratory of Tumor Biological Behaviors, Zhongnan Hospital of Wuhan University, Wuhan 430071, Hubei, China; j Hubei Cancer Clinical Study Center, Zhongnan Hospital of Wuhan University, Wuhan 430071, Hubei, China

**Keywords:** Gene delivery, Poly (beta-amino ester), Glutathione, ROS, Nanoparticle

## Abstract

**Introduction:**

CRISPR/Cas9 gene editing technology has significantly advanced gene therapy, with gene vectors being one of the key factors for its success. Poly (beta-amino ester) (PBAE), a distinguished non-viral cationic gene vector, is known to elevate intracellular reactive oxygen species (ROS) levels, which may cause cytotoxicity and, consequently, impact gene transfection efficacy (T.E.).

**Objectives:**

To develop a simple but efficient strategy to improve the gene delivery ability and biosafety of PBAE both *in vivo* and *in vitro*.

**Methods:**

We used glutathione (GSH), a clinically utilized drug with capability to modulating intracellular ROS level, to prepare a hybrid system with PBAE-plasmid nanoparticles (NPs). This system was characterized by flow cytometry, RNA-seq, Polymerase Chain Reaction (PCR) and Sanger sequencing *in vitro*, and its safety and efficacy *in vivo* was evaluated by imaging, PCR, Sanger sequencing and histology analysis.

**Results:**

The particle size of GSH-PBAE-plasmid NPs were 168.31 nm with a ζ-potential of 15.21 mV. An enhancement in T.E. and gene editing efficiency, ranging from 10 % to 100 %, was observed compared to GSH-free PBAE-plasmid NPs in various cell lines. In vitro results proved that GSH-PBAE-plasmid NPs reduced intracellular ROS levels by 25 %–40 %, decreased the total number of upregulated/downregulated genes from 4,952 to 789, and significantly avoided the disturbance in gene expression related to cellular oxidative stress-response and cell growth regulation signaling pathway compared to PBAE-plasmid NPs. They also demonstrated lower impact on the cell cycle, slighter hemolysis, and higher cell viability after gene transfection. Furthermore, GSH hybrid PBAE-plasmid NPs exhibited superior safety and improved tumor suppression ability in an Epstein–Barr virus (EBV)-infected murine tumor model, *via* targeting cleavage the EBV related oncogene by delivering CRISPR/Cas9 gene editing system and down-regulating the expression levels. This simple but effective strategy is expected to promote clinical applications of non-viral vector gene delivery.

## Highlights

• Glutathione (GSH) hybrid strategy is simple and effective in modulating intracellular ROS level and reducing gene expression disturbance induced by poly (beta-amino ester) (PBAE).
• GSH hybrid strategy enhances the transfection efficiency and gene editing efficiency of PBAE in various cell lines.
• GSH-PBAE-plasmid NPs have lower impact on the cell cycle, slighter hemolysis, and higher cell viability than PBAE-plasmid NPs.
• GSH-PBAE-plasmid NPs have a longer retention time in tumor, further improving the retention ability of plasmids.
• GSH-hybrid strategy improves therapeutic genes delivery *in vivo* and has a good therapeutic effect in xenograft tumor model.

## Graphical abstract

GSH hybrid PBAE-plasmid NPs deceased intracellular ROS generation induced by cationic polymer PBAE, thereby alleviating oxidative stress, minimizing gene expression disturbance, and thus reducing cytotoxicity and enhancing gene delivery.

**Figure:**
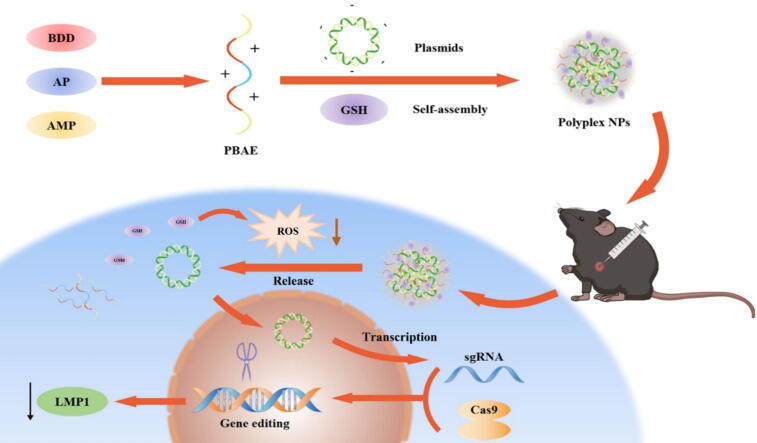

## Introduction

With the emergence of the CRISPR/Cas9 gene editing technology, gene therapy has gradually become an important part in treating various genetic diseases and tumors [1], [2], [3], [4]. Because of the physiological barriers and nucleases in the body, it is necessary to develop delivery vectors to successfully deliver gene drugs to target sites [5], [6]. Currently, viral and non-viral vectors are commonly used for gene delivery [7], [8]. Although viral vectors have excellent transfection capabilities, their applications are limited by their immunogenicity, carcinogenicity, insertion, and integration of host DNA, and their side effects [9], [10], [11]. Non-viral vectors, especially cationic polymers, possess the advantages of high gene loading capacity, easy preparation, and low immunogenicity, including poly (L-lysine) (PLL), polyethyleneimine (PEI), and poly (beta-amino ester) (PBAE) [12], [13], [14]. PBAE is a cationic polymer gene carrier with characterization of biodegradation, low toxicity, simple synthesis, and easily structural modification [15]. Positively charged PBAE compresses negatively charged DNA to a suitable size, facilitating its entry into cells, protecting it from degradation by nucleases, and thus achieving efficient DNA transfection [16].

However, cationic polymers have some side effects on cells owing to their potential to induce cell hemolysis, damage cell membranes, induce oxidative stress, affect DNA synthesis and gene expression, and affect cell metabolism [17], [18], [19], [20]. In particular, excessive reactive oxygen species (ROS) in cells stimulated by cationic polymers is an important issue, which in turn activates a series of cellular signaling pathways and affects cell proliferation, growth, and gene transduction efficiency [21], [22], [23], [24], [25], [26]. To mitigate the adverse effects induced by ROS, Paritosh *et al.* used chemical polymerization to insert small molecular antioxidants (quercetin and curcumin) into the main chain of PBAE materials to form antioxidant biopolymers [27]. When the materials degraded, they released active antioxidants that can scavenge free radicals, reduce oxidative stress, and improve biocompatibility. In addition, researchers have found that attachment of thioketone (TK) linkers or thioether dendrimers to PEI through disulfide bonds can not only lower intracellular ROS levels, but also enhance gene transfection efficiency (T.E.) and attenuate cellular toxicity [26], [28]. However, this strategy of cationic polymers requires precise reaction control conditions and may result in the formation of several side products, hindering the manufacturability and application of this approach.

Glutathione (GSH), a small molecular antioxidant in human body, is known for its antioxidant and anti-inflammatory effects. Various pharmaceuticals with GSH as a key ingredient have been developed and marketed. Because of the mechanism by which GSH reduces intracellular ROS levels and mitigates the cytotoxicity of drug delivery systems, it’ll be quite valuable to induce GSH into the cationic gene delivery system and reveal the possibility and mechanism to affect the gene transfection process.

**Fig. 1:**
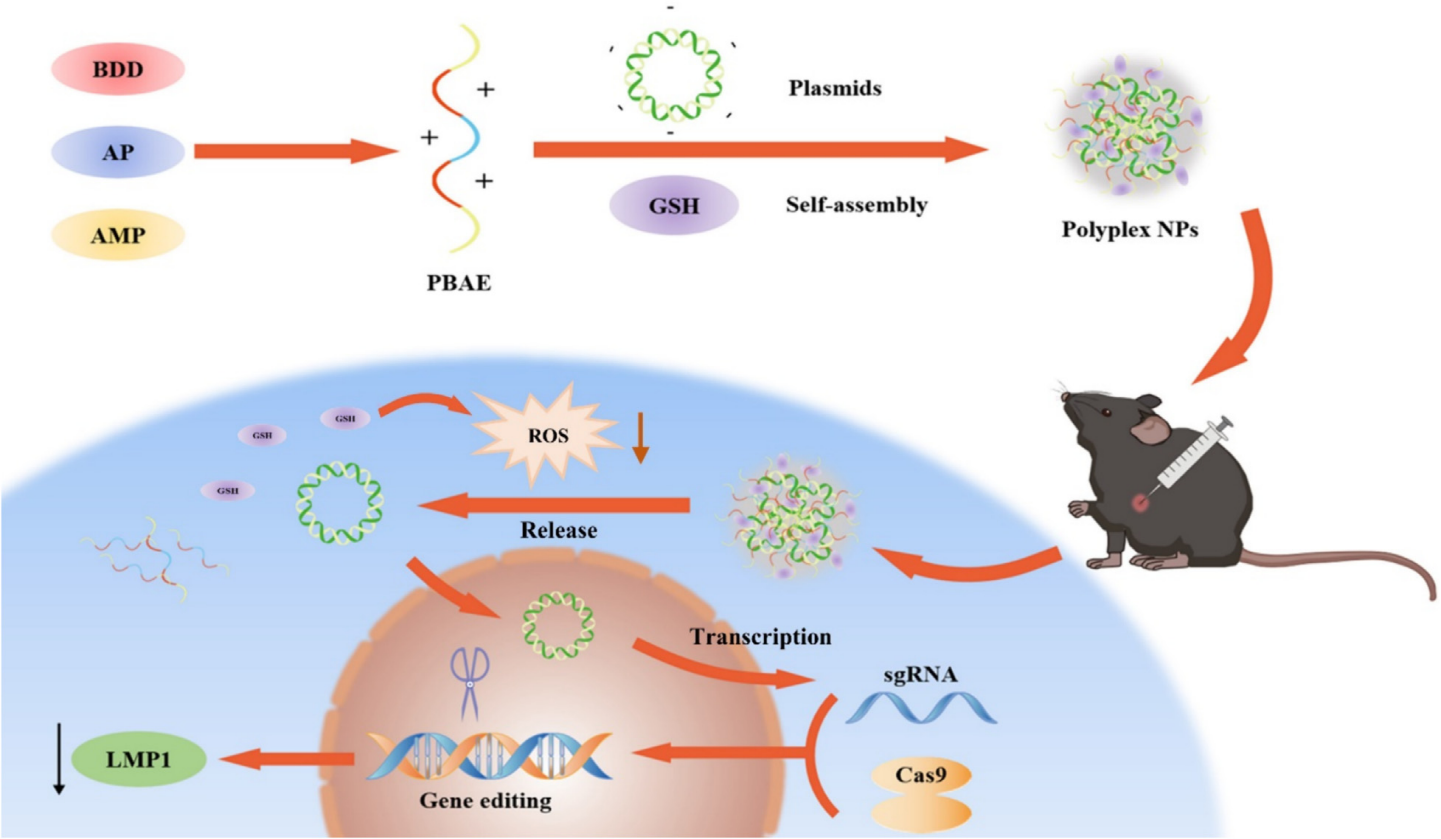
Fabrication of GSH-PBAE-plasmid NPs and the scheme for gene therapy. The GSH-PBAE-plasmid NPs were first prepared *via* electrostatic interactions. After endocytosis, the NPs release GSH to balance intracellular ROS concentration induced by PBAE. After transcription and translation, the CRISPR/Cas9 system targeting edits the LMP1 oncogene to inhibit the growth of tumor cells.

Here, we propose a minimalist gene delivery strategy, specifically, GSH hybrid PBAE-plasmid NPs (GSH-PBAE-plasmid NPs) (Fig. 1). In the hybrid NPs, the negatively charged GSH carboxyl groups combined with the amine group in PBAE through electrostatic interactions to achieve hybridization. The sulfhydryl group of GSH serves to reduce intracellular ROS, thereby alleviating oxidative stress, mitigating the disturbance of host cell gene expression following the entry of cationic polymer NPs, and enabling cells to revert to their normal physiological state, thus resulting in reduced cytotoxicity and enhanced gene delivery both *in vitro* and *in vivo*.

## Materials and methods

### Materials, cells and animals

The 5-Amino-1-pentanol (AP) and 1,4-butanediol diacrylate (BDD) were purchased from TCI (Shanghai, China). The 1-(3-aminopropyl)-4-methylpiperazine (AMP) was from Adamas (Shanghai, China). Polyethyleneimine (PEI 25KD) was obtained from Sigma-Aldrich, and chlorin e6 (Ce6) was purchased from Frontier Scientific Corporation. Glutathione (GSH) was purchased from Aladdin (Shanghai, China). The plasmids expressing green fluorescent protein (GFP) (5 K/10 K/13 K bp), plasmid expressing mCherry protein (mCherry10Kbp), plasmid expressing luciferase reporting gene, CRISPR/Cas9 plasmids [latent membrane protein-sgRNA 4 plasmid (LMP-g4), Mucin 2 plasimid (MUC2P), Lysine Acetyltransferase 6A plasimid (KAT6A-sg1) and oligodeoxynucleotide (ODN), were all from Generulor Co., Ltd., Wuhan (China). The HEK 293 T cells (human embryonic kidney cells) and C666-1 cells (Epstein–Barr virus (EBV) −positive nasopharyngeal carcinoma cells, stably expressing LMP1) purchased from Guangzhou Xinyuan Biotechnology Co., Ltd.. 293-L (HEK 293 T cells stably expressing LMP1) and SCC-7-L (mouse head and neck squamous cell carcinoma cells stably expressing LMP1) were constructed by Generulor Co., Ltd., Wuhan (China). HEK 293 T and 293-L cells were cultured in high glucose dulbecco's modified eagle medium (DMEM) (Gibco, USA) supplemented with 10 % fetal bovine serum (FBS) (Tian Hang Biotechnology Co. Ltd, China) and 1 % penicillin/streptomycin (Thermo Fisher, USA). C666-1 and SCC-7-L were cultured in Roswell Park Memorial Institute (RPMI) 1640 (Gibco, USA) complete medium. C57 mice (female, 14–16 g) and balb/c nude mice (male, 18–20 g) were purchased from Wuhan Shibei Biotechnology Co., Ltd.

### Synthesis and characterization of PBAE

**Fig. 2:**
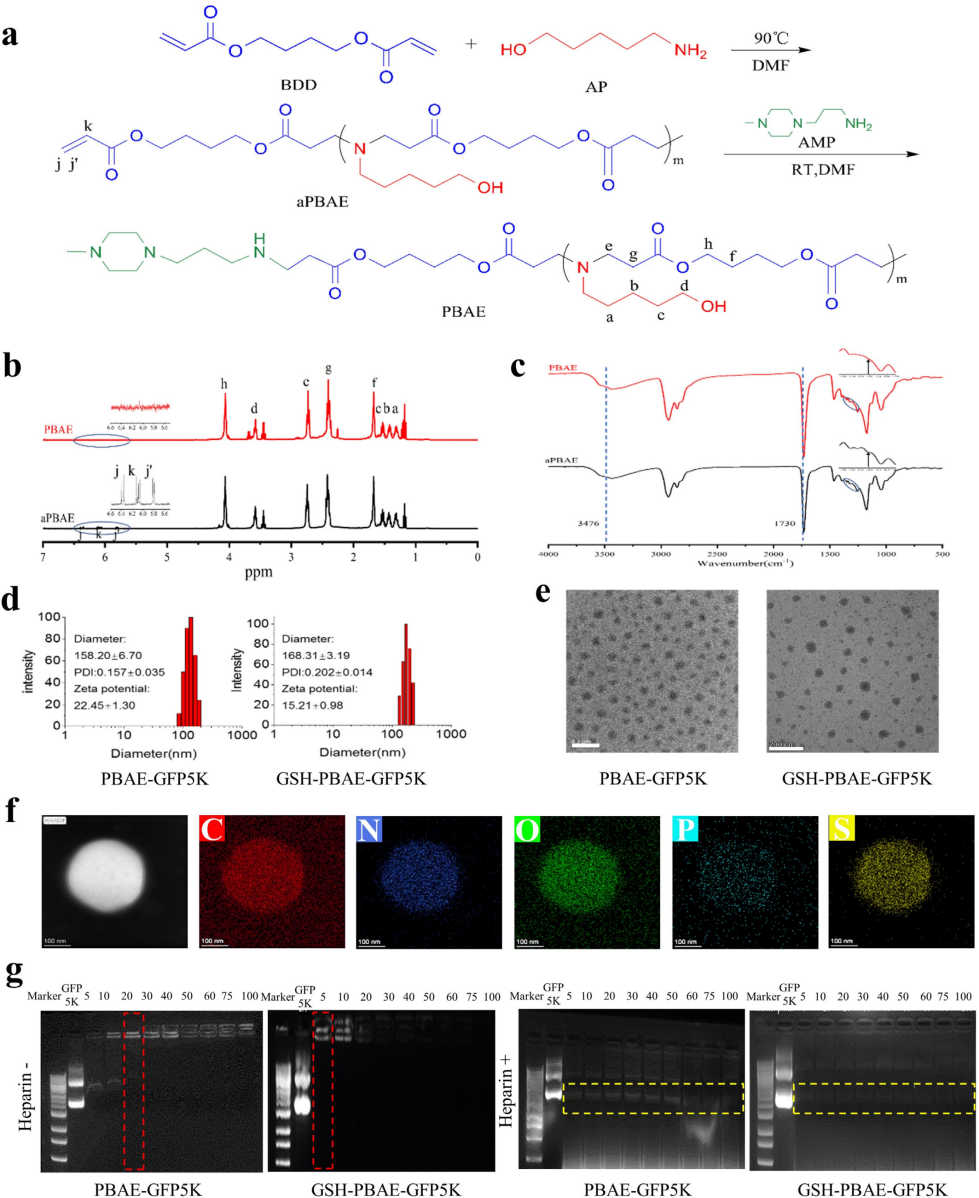
Characterization of GSH-PBAE-plasmid NPs: (a) Synthesis scheme of PBAE. (b) ^1^H NMR spectra of PBAE and aPBAE. (c) FTIR spectra of PBAE and aPBAE. (d) Particles size and ξ-potential and (e) TEM (Scale bar: 200 nm) of PBAE-GFP5K NPs and GSH-PBAE-GFP5K NPs. (f) FTEM of GSH-PBAE-GFP5K NPs (Scale bar: 100 nm). (g) Agarose gel electrophoresis pattern of PBAE-GFP5K NPs and GSH-PBAE-GFP5K NPs at different mass ratios before (−) and after (+) Heparin treatment; Marker: 1 kb, GFP: free GFP solution, red box: the mass ratio at which no GFP band was observed, and yellow box: GFP band (released from polyplex NPs due to the replacement by Heparin).

The synthesis of Poly (β-amino ester) was carried out in two steps (Fig. 2a). The first step was the synthesis of aPBAE (intermediate product), which contains double bond acrylate end group, by dissolving BDD and AP in N, N-dimethylformamide (DMF) at a molar ratio of 1:1 and reacting at 90 °C for 36 h. The second step was to synthesize the final product (PBAE) by dissolving aPBAE in DMF, adding 5 times the molar amount of AMP, and reacting at room temperature for 36 h. Following each stage of the reaction, the polymers were precipitated with diethyl ether, washed by diethyl ether twice and dried under vacuum. PBAE and aPBAE were characterized by Hydrogen-nuclear magnetic resonance (^1^H NMR) (400 MHz) (Bruker, Switzerland) and Fourier transform infrared spectroscopy (FT-IR) (wavelength range 400–4000 cm^−1^) (PerkinElmer, USA).

### Preparation and characterization of polymer-plasmid NPs

To prepare PBAE and PEI-plasmid NPs, PBAE was dissolved in acid buffer (pH 5.0) at 15 μg/μL. The polymer solution and plasmids were mixed according to different mass ratios, then incubated at room temperature for 30 min. Different GFP plasmids (5 K, 10 K and 13 K bp) were used to study the mass ratios of polymer/plasmid (PBAE group: 30:1, 50:1, 60:1, 75:1, 100:1 and PEI group: 1.6:1, 3.2:1, 4.8:1, 6.4:1, 9.6:1) to obtain the optimal ratio of PBAE/PEI-plasmid. To prepare GSH-PBAE-plasmid NPs, PBAE-plasmid NPs were formulated under the optimal ratio (60 μg PBAE: 1 μg plasmid, Figure S1). After incubation at room temperature for 10 min, GSH (360 μg) was added and the solution was incubated for another 20 min. Succinic acid (C_4_H_6_O_4_) hybrid PBAE-plasmid NPs (C_4_H_6_O_4_-PBAE-plasmid NPs) were prepared in the same way as a control.

The particle sizes and ζ-potentials of PBAE or GSH-PBAE-plasmid NPs were measured by dynamic light scattering (DLS, ZetaPALs, Brookhaven, USA), and the stability of NPs in FBS over 24 h was investigated at room temperature. The morphology and elemental composition of the GSH-PBAE NPs were observed using transmission electron microscope (TEM, Tecnai G2-20, FEI, Netherlands) and field emission transmission electron microscopy (FTEM) (Talos F200X, FEI, Netherlands), respectively. Furthermore, the gene loading capacity of NPs was assessed by agarose gel electrophoresis with different polymer-plasmid ratios (5: 1, 10: 1, 20: 1, 30: 1, 40: 1, 50: 1, 60: 1, 75: 1 and 100: 1). The NPs solution was also treated with heparin (12.5 mg/mL) for 30 min to observe the release of the plasmids.

### Transfection efficiency

PBAE-plasmid NPs with different mass ratios (30:1, 50:1, 60:1, 75:1 and 100:1) were prepared as described above. The cells (293 T, 293-L, SCC-7-L, MCF-7 and Raw264.7) were seeded into six-well plates at a density of 3 × 10^5^ cells per well. When the cell grew to 70 % −80 % of density, 2 ml of serum free medium to replace the complete medium, then then NPs (2 μg plasmids/well) were added into corresponding well to culture 4 h, then the treated cells were cultured in complete medium for 2 h −72 h, according to the research purpose to select the culture time, and non-treated cells were controls. GFP and PEI 25 kD-plasmids NPs of were prepared and added simultaneously as positive controls. The treated cells were observed under an inverted fluorescence microscope (Olympus, Japan or NOVEL, China), or analyzed by flow cytometry (Accuri C6, BD, USA, or LE-SH800SDP, Sony, Japan) to detect the T.E..

### Cellular uptake studies

*Uptake.* PBAE labeled with Ce6 was used in the NPs, and then delivered the NPs (1 μg plasmids/well) to HEK 293 T cells in twelve-well plates with endocytosis inhibitors [29]. After treated cells for 0.5, 1, 2, and 4 h respectively, cells were fixed with 4 % para formaldehyde solution (300 μL/well) then stained by DPAI (200 μL/well). Each cell slide was sealed by antifade mounting medium (10 μL) and observed under laser scanning confocal microscopy (Sangon Biotech, China) to obtain the uptake efficacy of the NPs, or just collected the cells to analyze by flow cytometry (BD, USA).

*Uptake mechanism.* The HEK293T cells in six-well plates were preprocessed with three kinds of endocytosis inhibitor, including colchicine (microtubule-assisted endocytosis inhibitors, 5 × 10^−6^ M)(microtubule-assisted endocytosis inhibitors) [30], nystatin (small-mediated inhibitor, 20 × 10^−6^ M) and chlorpromazine (clathrin-mediated endocytosis inhibitor, 28 × 10^−7^ M) [31], and these cells incubated with the polymer-plasmids NPs (1 μg plasmids/well) at 37 °C for 30 min, then discarded inhibitor solution and added the NPs to culture for 4 h in serum free medium. The cell uptake at 4 °C was performed in the similar way. The non-treated cells (blank cells) and non-inhibitor group were controls. The cells in each group were collected for flow cytometry analysis (BD, USA).

### Intracellular ROS assays

The intracellular ROS level was detected using the fluorescent probe 2,7-dichlorodi hydro fluorescein diacetate (DCFH-DA) according to the instructions of the ROS detection kit (Solarbio, CA1420), and the Rosup as a positive control. HEK 293 T cells were seed to a twelve-well plates and incubated with NPs (1 μg plasmids/well) to culture in serum free medium for 0.5 h, 1 h, 2 h, 4 h, and 8 h, and non-treated cells group as a control. Then, detected the fluorescence intensity of DCF using the inverted fluorescence microscopy (485 nm, Olympus, Japan) and the treated cells were collected to analyze by a flow cytometer (BD, USA).

### Rna-seq transcriptomics

HEK 293 T cells treated with different polymers-plasmid NPs after 36 h co-incubation were collected. Then the total RNA was extracted with Trizol reagent (Thermo, 15596026) and the mRNA were isolated using poly-T oligos. The mRNA sequencing libraries were prepared with VAHTS Universal V10 RNA-seq Library Prep kit (Vazyme, NR606-01) following manufacturer’s instructions. The libraries were subjected to 150-bp pair-end sequencing with HiSeq Xten systems at Generulor Co., Ltd. (Zhuhai, China). Sequencing adapters and low-quality bases were removed with fastp using default parameters. Mapping to human reference genome hg38 were carried out using subread package. TPM designated gene expressions were quantified with StringTie against National Center for Biotechnology Information (NCBI) Refseq annotations.

### In vitro biological studies

*Cell cycle influence assays.* HEK 293 T cells were treated with different NPs and then collected the cells after 36 h co-incubation (six-well plates, 2 μg plasmids/well). According to the instructions of the Cell Cycle Analysis Kit (Beyotime, C1052), PI was used to stain the cells, and the influence of the different polymers-plasmids NPs on the cell cycle was evaluated using a flow cytometer (BD, USA).

*MTT assays.* HEK 293 T cells were seeded in 96-well plates for culturing an appropriate density. The non-treated cells well as a negative control, and the empty well as a blank control. The stock NPs solution was diluted with medium to prepare test solutions, which were added into the wells (0.1 μ g plasmids/well). After culturing for 36 h, the medium dilution (0.5 mg/ml, 100 μL) was added to replace the medium in each well and continued to culture for 2 h. Then, the Dimethyl sulfoxide (DMSO) solvent was to replace the 3-[4,5-dimethylthiazol-2-yl]-2,5 diphenyl tetrazolium bromide (MTT) dilution for 10 min of incubation at 37 °C. The absorbance was measured by a microplate reader (490 nm). Cell survival rate = (As-Ab)/(Ac-Ab) × 100 % (As: absorbance of the experimental well, Ac: average absorbance of the negative control well, Ab: absorbance of the blank control well).

### In vitro gene editing efficiency studies

The *MUC2* gene and *KAT6A* gene were the selected as targets in HEK 293 T cells, while in 293-L cells, it’s the *LMP1* gene. The Crispr-Cas9 plasmids specifically designed to these target gene were used, and information of sgRNA, PAM and primers were listed in Table S1 and S2. To evaluate gene editing efficiency, the treated cells (six-well plates, 2 μg plasmids/well) were collected after 36 h, and extracted the DNA using the DNA extraction kit (Transgen, EE101-01). Gene editing of *KAT6A* was studied by Sanger sequencing analysis. The genomic regions flanking the target sites were amplified using the corresponding primers. Polymerase chain reaction (PCR) products were prepared and sequenced using Sanger sequencing.

For *MUC2*, the genomic regions adjacent to the target sites were specifically amplified using T7E1 primers and the Q5® Hot Start High Fidelity 2 × Master Mix, provided by New England Biolabs in Beijing, China. The PCR-derived products were meticulously purified through the application of KAPA Pure Beads. Subsequently, 200 ng of each purified PCR product was subjected to denaturation via heating, followed by reannealing to create heteroduplex DNA. This reannealed sample was then incubated with 10 units of T7 Endonuclease I (New England Biolabs) for 15 min at 37 °C. Finally, the disruption of *MUC2* was confirmed by electrophoresis on a 2 % TAE-agarose gel, pre-stained with ethidium bromide.

Gene editing of *LMP1* was then checked by the ODN-PCR method [32]. The polymer–plasmids/OND NPs were delivered to 293-L cells. The genomes of 293-L cells were extracted using a DNA extraction kit. Gene editing was then detected by assessing the amplification of ODN fragments within PCR products as ODN could be stochastically integrated into the double-strand breaks (DSBs) induced by the cleavage process.

### *In vivo* studies

*Biodistribution.* The healthy C57 mice were injected subcutaneously with SCC-7-L cells in the right axillary skin to form tumor (50–100 mm^3^). Then the mice were randomly divided into 4 groups (3 mice per group) as follows: the free Ce6 group, the Ce6/PEI-plasmid (LMP-g4) NPs group, the Ce6/PBAE-LMP-g4 NPs group, and the Ce6/GSH-PBAE-LMP-g4 NPs group. Ce6 labelled PBAE and PBAE (1:3) were used to prepare NPs for *in vivo* tracking. The polymer-plasmids NPs (15 μg plasmids) were peritumorally injected and the mice were narcotized after 0.5 h, 1 h, 2 h, 4 h, 8 h, 12 h and 24 h to observe the accumulation and retention of NPs in the *in vivo* imaging system (Pearl Imager, LICOR, USA). Finally, the mice were sacrificed after 24 h to collect the tumors and major organs (heart, liver, spleen, lung, and kidney) and analyzed them using the *in vivo* imaging system. HEK 293 T cells cluster model was also built to evaluate the biodistribution of the NPs (detailed in Supplementary Information).

To further investigate the distribution of gene editing systems in mice, SCC-7-L tumor bearing mouse model (50–100 mm^3^) was established as described above, and the mice were randomly divided into 4 groups (3 mice per group), with peritumoral injection of GFP, PEI-GFP NPs, PBAE-GFP NPs, and GSH-PBAE-GFP NPs (15 μg plasmids/mouse) on day 5, day 7, day 9, and day 11. On day 12, the tumors, blood and major organs (heart, liver, spleen, lung and kidney) were collected. The total DNA of the samples was extracted by Genomic DNA Kit (TransGen, EE101-11). For PCR analysis, 20 ng DNA of each sample was used and 1 ng GFP was chosen as positive control. Each sample was cycled 32 times at 95 °C for 15 s followed by 60 °C for 15 s and then up to 72 °C with 60 S/kb. The final extension time is 5 min at 72 °C. After PCR amplification, the samples were detected by gel electrophoresis. The tumor volume and mice body weight were also monitored to evaluate the effects of the NPs on tumor growth and mice.

*Growth inhibition of EBV-infected tumors.* The mice were injected with SCC-7-L cells on Day 0 to form tumors and divided into 5 groups (5 mice per group) as follows: the saline group, the plasmid (LMP-g4) group, the PEI-LMP-g4 NPs group, the PBAE-LMP-g4 NPs group and the GSH-PBAE-LMP-g4 NPs group. The NPs (15 μg plasmids) were injected on day 5, day 7, day 9, and day 11. The tumor volume and mice body weight were synchronously monitored. The tumor growth coefficient = Vt/V0 (V_0_: the tumor volume at initial administration, Vt: the tumor volume at end of the experiment), and the tumor growth inhibition rate was calculated as [1-tumor weight (experimental group)/ tumor weight (control group)] × 100 %. The tumors, major organs (heart, liver, spleen, lung, and kidney) were dissected, weighed, and photographed on day 12. The total RNA of tumor tissue was extracted using Trizol reagent and reverse-transcribed to cDNA and then qPCR was performed. The gene expression level in each sample was normalized to the GAPDH expression level, and the relative expression level of RNA was calculated using the comparative Ct method. To further evaluate the *in vivo* gene editing efficacy, the SCC-7-L tumor bearing mouse model with tumor size of 50–100 mm^3^ was established as described above, and the mice were randomly divided into 4 groups (3 mice per group), with peritumoral injection of LMP-g4, PEI-LMP-g4 NPs, PBAE-LMP-g4 NPs and GSH-PBAE-LMP-g4 NPs (15 μg plasmids/mouse). After 24 h, the mice were sacrificed and the tumors were collected. The total DNA of the samples was extracted by Genomic DNA Kit (TransGen, EE101-11), amplified by PCR, and detected by Sanger sequencing.

*Safety evaluation.* The blood was collected from the eyes of mice on day 12. The content of alanine aminotransferase (ALT) and blood urea nitrogen (BUN) were measured using a blood biochemical detection kit (Solarbio, BC1555and BC1535). Subsequently, the mice were sacrificed, and the organs were fixed with 4 % paraformaldehyde, followed by embedding in paraffin, sectioning, dewaxing, staining with Hematoxylin-eosin (H&E), rinsing, and observed by microscope.

### Statistical analysis

All quantitative data expressed as mean ± standard deviation (SD) was measured at least three times in parallel, and analyzed using one-way ANOVA or Student’s *t*-test in GraphPad Prism software (version 7.00), with p <0.05 indicating significant differences.

### Ethics statement

All the *in vivo* experiments were managed by Myhalic biotechnology Co. Ltd. (Wuhan, China), the Institutional Animal Care and Use Committee (IACUC) number was HLK-20231110-001, and in accordance with the regulations of Chinese law, and approved by the Experimental Animal Ethics Committee of the company.

## Results

### Synthesis and characterization of PBAE

The PBAE polymer was synthesized in two steps (Fig. 2a), and the chemical structures of PBAE and aPBAE were confirmed by ^1^H nuclear magnetic resonance (^1^H NMR, Fig. 2b) and Fourier transform infrared (FT-IR) spectroscopy (Fig. 2c). In the ^1^H NMR spectra, the hydrogen signal peaks of –CH_2_CH_2_COO- at 2.73 ppm (e) and 2.41 ppm (g) were formed after the reaction of the acrylate bond in the BDD with the amine group in the AP. The acrylate bond peaks at 5.80 ppm (jʹ), 6.37 ppm (j), and 6.09 ppm (k) in aPBAE were not apparent for PBAE, which indicated the successful synthesis of PBAE. The FT-IR spectra demonstrated a stretching vibration peak of the carbonyl group on BDD with a narrow and sharp absorption peak at 1730 cm^−1^. Additionally, the peak at 1283 cm^−1^, which appeared only for aPBAE, indicates the stretching vibration peak of the asymmetric carbon–carbon double bonds, further confirming the synthesis of PBAE.

### Formation and characterization of GSH-PBAE-plasmid hybrid NPs

To prepare the GSH-PBAE-plasmid hybrid NPs with proper compositions, the ratios of PBAE plasmids were first evaluated in 293 T cells (Figure S1). The T.E. of the PBAE-GFP5K NP group reached over 97 % at ratios of 30:1–100:1, whereas the highest T.E. for the positive control, PEI-GFP5K, was only 74.8 % at a ratio of 4.8:1, indicating that the gene delivery ability of PEI was much lower than that of PBAE. It’s also found that the T.E. decreased when larger plasmids (GFP10 K, GFP13 K) were used. The highest T.E. of PBAE-GFP10K NPs (73.6 %) and PBAE-GFP 13 K NPs (68.4 %) were both at a ratio of 60:1, and the highest T.E. of PEI-GFP10K NPs (35.3 %) and PEI-GFP13K NPs (27.2 %) were all at a ratio of 4.8:1 (Figure S1). These ratios were used in subsequent experiments. Moreover, for both PBAE-GFP5K NPs and PEI-GFP5K NPs, the T.E. reached the highest values at 36 h (97.3 % for PBAE, 75.8 % for PEI) in 293 T cells (Figure S2). So 36 h was set as the transfection time for the subsequent experiments.

GSH-PBAE-plasmid hybrid NPs and PBAE-plasmid NPs were prepared at the optimal mass ratio (60:1). The particle size of the GSH-PBAE-GFP5K NPs (168.31±3.19 nm) was similar to the PBAE-GFP5K NPs (158.20±6.70 nm) with a narrow size (Polydispersity Index, PDI ≤0.2). The ζ-potential of the GSH hybrid PBAE-plasmid NPs decreased slightly from 22.45±1.30 mV (PBAE-plasmid NPs) to 15.21±0.95 mV, maybe due to the influence of negative charged COO^–^ group in GSH (Fig. 2d). These NPs were uniform nano-sized spherical particles observed by TEM with ∼100 nm in size, which was smaller than the DLS-measured size owing to dehydration induced volume shrinkage (Fig. 2e). To further confirm the formation of hybrid structure, FTEM was utilized and the elements were evenly distributed with good co-localization in the polymer (N), plasmid (P), and GSH (S), indicating the successful preparation of GSH-PBAE-plasmid NPs (Fig. 2f). During the 24-h storage period in FBS, the particle size of PBAE-GFP5K NPs increased slightly after 8 h, whereas the GSH-PBAE-GFP5K NPs remained stable (Figure S3). Agarose gel electrophoresis results of NPs with various ratios (5:1–100:1, Fig. 2g) demonstrated that for PBAE-GFP5K NPs, a mass ratio of at least 20:1 was required to ensure the absence of visible plasmid bands. However, for GSH-PBAE-GFP5K NPs, a ratio of only 5:1 was needed, indicating the improvement of plasmids encapsulation by GSH hybrid strategy. Furthermore, the addition of heparin resulted in the presence of GFP5K plasmid bands in both types of NPs, indicating that GSH hybridization did not affect plasmid release from the NPs in the cytoplasm.

### GSH hybrid strategy improved transfection efficacy *in vitro*

**Fig. 3:**
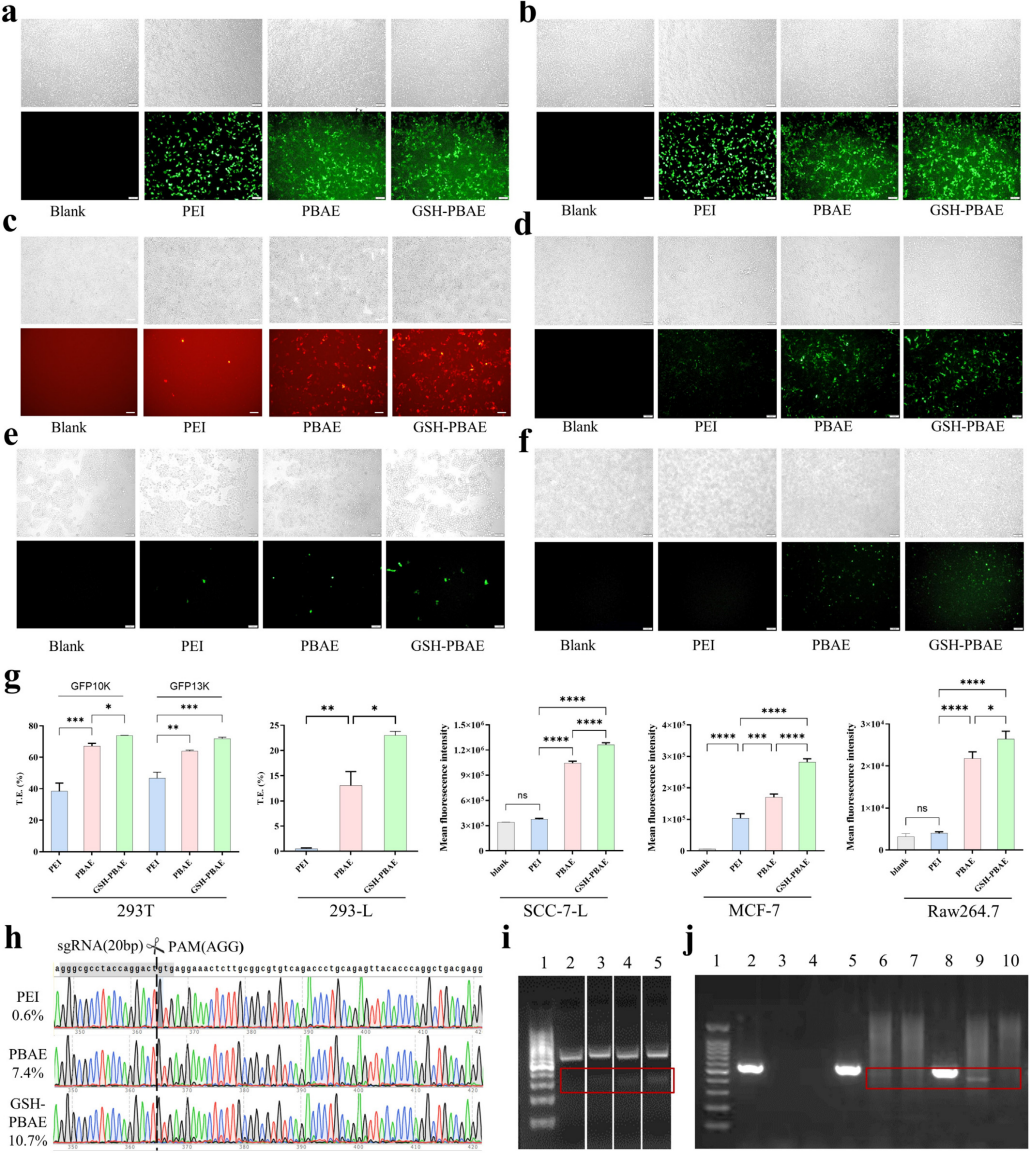
T.E. and gene editing efficiency of different polymers-plasmids NPs in various cell lines. Typic fluorescence microscopy (Scale bar: 100 μm) of (a) GFP 10 K and (b) GFP 13 K in 293 T cells, (c) mCherry 10 k in 293-L cells, (d) GFP 5 K in SCC-7-L cells, (e) GFP 10 K in MCF-7 cells and (f) GFP 10 K in Raw264.7 cells. (g) Quantitative cytometric analysis of gene transfection efficacy in the corresponding cells. (h) The gene editing results of sanger sequences of PCR products (KAT6A gene) after delivering KAT6A-sg1 plasmids in HEK 293 T cells. (i) The results of T7E1 assay after delivering MUC2P in 293 T cells (1: 1 K bp maker, 2: blank, 3: PEI-plasmids NPs, 4: PBAE-plasmid NPs, 5: GSH-PBAE-plasmid NPs). (j) The gene editing results of DNA gel electrophoresis after co-delivery of LMP-g4 and ODN (1: 100 bp maker, 2: blank-I, 3: blank-II, 4: blank-III, 5: PBAE-LMP-g4-I, 6: PBAE-LMP-g4-II, 7: PBAE-LMP-g4-III, 8: GSH-PBAE-LMP-g4-I, 9: GSH-PBAE-LMP-g4-II, 10: GSH-PBAE-LMP-g4-III; primers: I: LMP-g4-F1 + LMP-g4-R1, II: LMP-g4F1 + ODN-R1, III: LMP-g4R1 + ODN-R1, Table S2) in 293-L cells. One-way ANOVA was for statistical analysis, *p <0.05, **p <0.01, ***p <0.001, ****p <0.0001, ns: no significant difference.

GFP plasmids (10 K and 13 K bp) and mCherry plasmids (10 K bp) were selected to evaluate the T.E. of the NPs in several cell lines. In HEK 293 T cells, the T.E. of GSH-PBAE-GFP10K/13 K NPs (73.8 %/71.9 %) were higher than that of PBAE-GFP10K/13 K NPs (67.2 %/64.1 %), which were all considerably higher than the “gold standard” PEI 25kD (Fig. 3a, b, g). Furthermore, in 293-L cells (Fig. 3c, g), the T.E. of GSH-PBAE-mCherry10K NPs (22.62 %) was also greater than that of PBAE-mCherry10K NPs (11.11 %) and considerably higher than that of PEI-mCherry10K (0.45 %). The transfection of SCC-7-L cells was challenging (Fig. 3d, g). However, GSH-PBAE NPs still exhibited advantages in T.E. improvement. The fluorescence intensity in the cells of PBAE and GSH-PBAE groups was significantly greater (P <0.0001) than that of the PEI 25KD group (no difference from the blank group). The fluorescence of the GSH-PBAE-GFP5K NPs was approximately 1.27 times that of the PBAE-GFP5K NPs. The same trend was also observed in MCF-7 cells and Raw 264.7 cells (Fig. 3e, f, g). The fluorescence of the GSH-PBAE-GFP10K NPs was approximately 1.65 times and 1.21 times these of the PBAE-GFP10K NPs, and was 2.71 times and 6.62 times these of the PEI 25KD group in MCF-7 cells and Raw 264.7 cells.

### GSH-PBAE-plasmid NPs had no impaction on the efficiency and mechanism of cells uptake

**Fig. 4:**
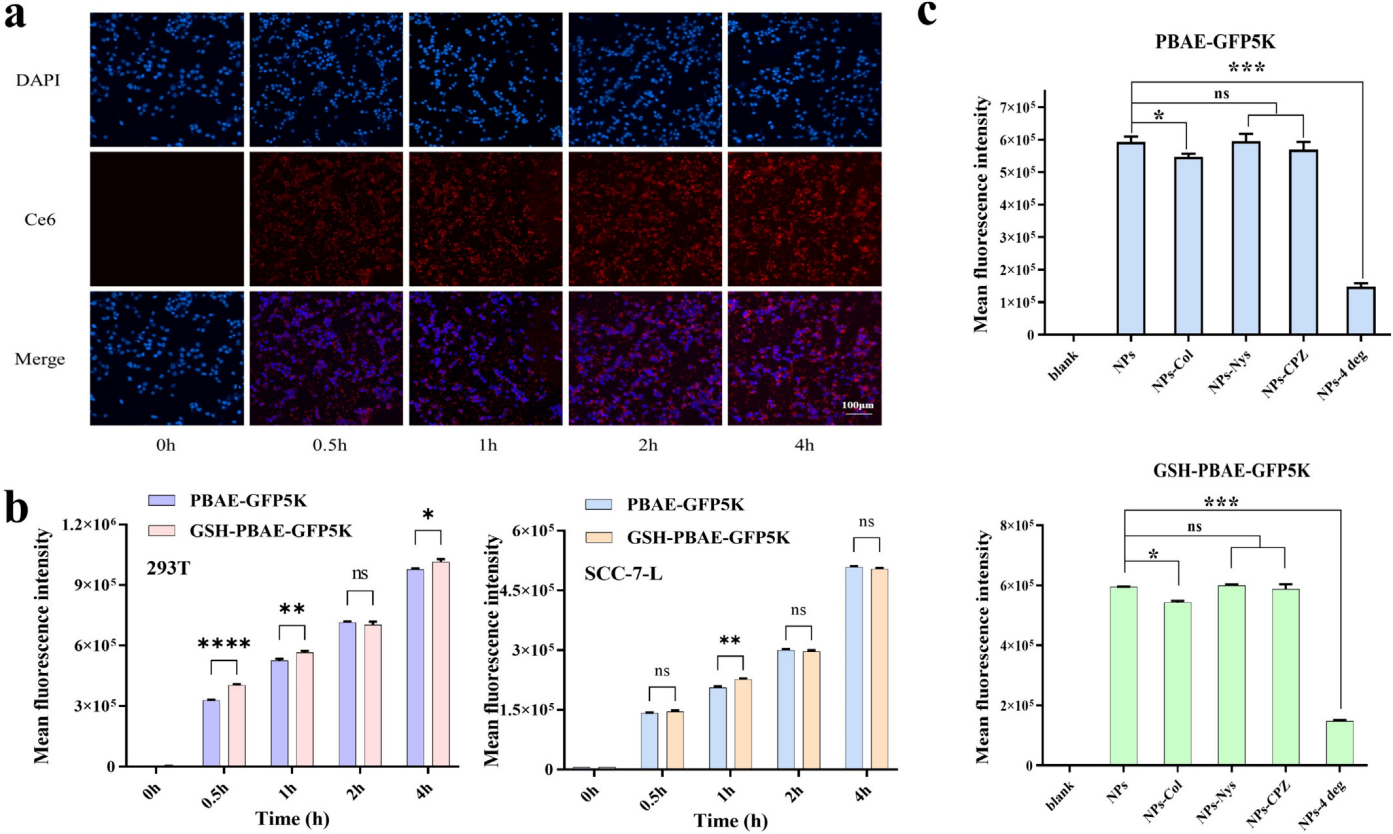
Intracellular uptake of GSH-PBAE-GFP5K NPs in SCC-7-L and HEK 293 T cells. (a) Typic confocal fluorescence microscopy in SCC-7-L cells at different time intervals (Scale bar: 100 μm). (b) Mean fluorescence intensity statistics of HEK 293 T cells and SCC-7-L cells incubated with PBAE-GFP5K NPs and GSH-PBAE-GFP5K NPs (n = 3). (c) Uptake mechanism of HEK 293 T cells incubated with different NPs. Mean fluorescence intensity statistics of HEK 293 T cells incubated with PBAE-GFP5K NPs and GSH-PBAE-GFP5K NPs (n = 3). Col: Colchicine, Nys: Nystatin, CPZ: Chlorpromazine hydrochloride, 4 deg: 4 °C. One-way ANOVA was for statistical analysis, ***p <0.001, **p <0.01, *p <0.05, ns: no significant difference.

Ce6-labeled PBAE were used to explore the NP uptake by SCC-7-L and HEK 293 T cells. For SCC-7-L cells, significant red fluorescence was observed at 0.5 h, and its intensity increased with time (Fig. 4a). The red fluorescence intensity at 4 h was approximately 17.2 times that at 0.5 h. In HEK 293 T cells, the average red fluorescence intensity at 4 h was approximately 146.5 times that at 0.5 h, indicating that both cell types could rapidly and efficiently take up the GSH-PBAE NPs (Fig. 4b). Quantitative results demonstrated that the average fluorescence intensities of PBAE and GSH-PBAE NPs were similar in both cell lines (Fig. 4b), indicating that GSH hybridization did not affect the cellular uptake ability of PBAE-plasmid NPs. In addition, after treatment with three different uptake inhibitors (colchicine, nystatin, and chlorpromazine hydrochloride) in HEK293T cells, the average fluorescence intensity of cells in the PBAE and GSH-PBAE groups only slightly decreased after colchicine treatment. However, both groups demonstrated significant decreases after culturing at 4 °C, with cell fluorescence intensity only 24.05 % and 24.89 % of that in the untreated NP group, respectively (Fig. 4c). This result indicated that the uptake of PBAE NPs and GSH-PBAE NPs was affected mainly by temperature. Most NPs entered the cells mainly through membrane fusion and a small portion through microtubule trafficking.

### GSH-PBAE-plasmid NPs reduced intracellular ROS levels

**Fig. 5:**
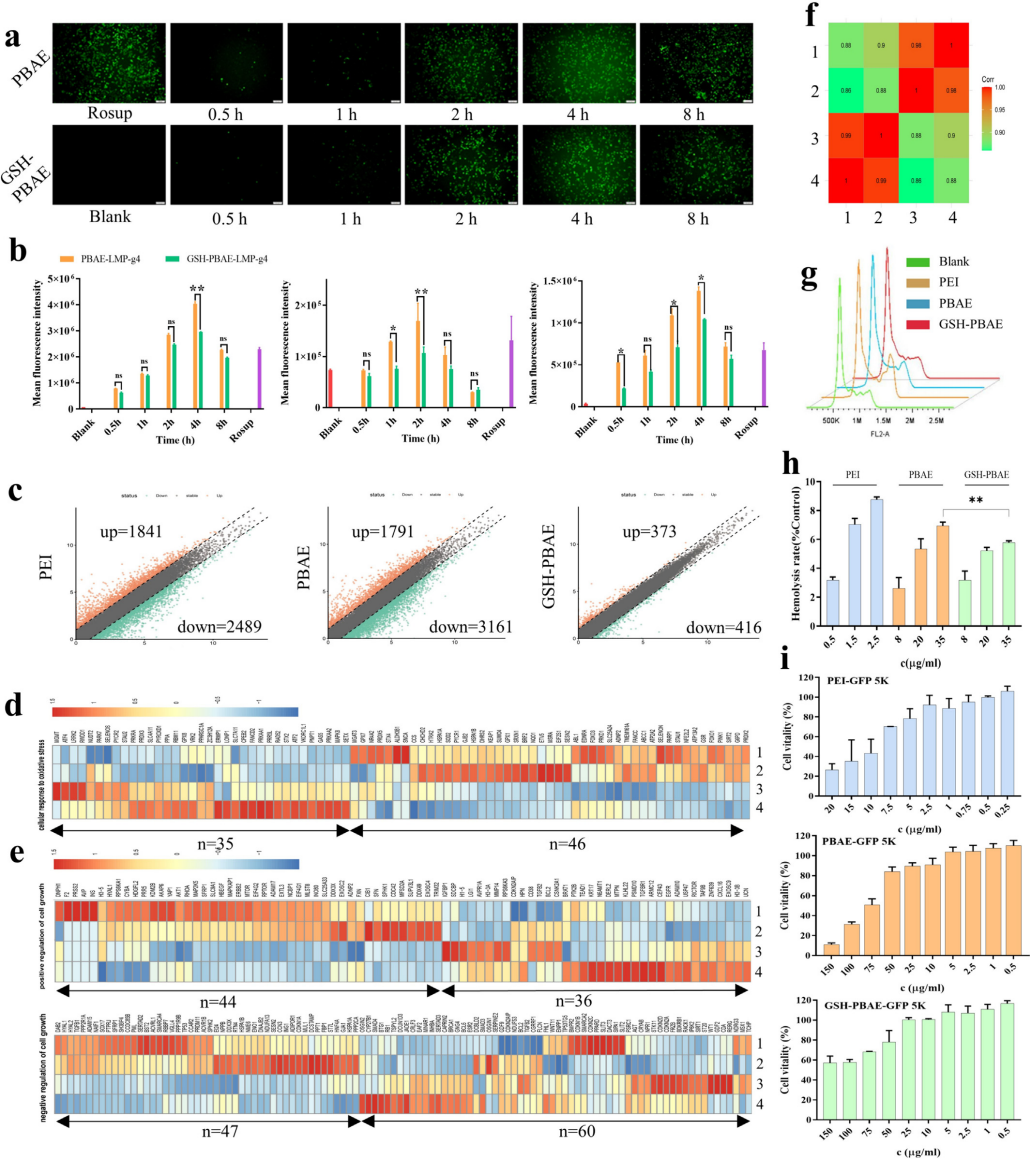
The evaluation of intracellular ROS level influence and biocompatibility of different NPs on cells. (a) Typic fluorescence microscopy pictures of detecting the intracellular ROS levels at different time intervals (Scale bar:100 μm) in HEK 293 T cells. (b) The results of mean fluorescence intensity statistics on HEK293T, SCC-7-L and C666-1 cells (n = 3). (c) Scattergram of gene expression in each cationic polymers-plasmids NPs treated cells group relative to the blank group (red indicates up-regulated expression, green indicates down-regulated expression, and gray indicates stable expression of genes, and the black dashed line indicates the set threshold: |log2FC|>1). Heat map of (d) cellular oxidation stress response gene expression and (e) positive and negative regulation of cell growth gene expression (1: PEI-GFP5K NPs, 2: PBAE-GFP5K NPs, 3: GSH-PBAE-GFP5K NPs, 4: blank). (f) Correlation of gene expression in each group (1: GSH-PBAE-GFP5K NPs, 2: blank, 3: PEI-GFP5K NPs, 4: PBAE-GFP5K NPs). (g) The migration diagram of HEK 293 T cell cycle after incubation with different NPs using LMP1-g4 plasmids. (h) The quantitative statistical hemolysis results of different NPs using LMP1-g4 plasmids (n = 4). (i) Cell viability of HEK 293 T cells incubated with PEI-GFP5K, PBAE-GFP5K and GSH/PBAE-GFP5K NPs (n = 3). One-way ANOVA was for statistical analysis, **p <0.01, *p <0.05, ns: no significant difference.

To verify whether GSH was effectively delivered by the NPs and played a role in reducing the intracellular ROS levels, GSH-PBAE-LMP-g4 and PBAE-LMP-g4 NPs were seeded into HEK 293 T, SCC-7-L, and C666-1 cells using the fluorescent probe DCFH-DA to detect the intracellular ROS levels. The fluorescence intensity of the three cell lines significantly increased after treatment with Rosup (positive control), indicating that oxidative stress conditions could lead to increased intracellular ROS levels (Fig. 5a, b). Following the delivery of these two NPs, the average fluorescence intensity in each cell line gradually increased, reaching its peak at 2 h (in SCC-7-L cells) or 4 h (in HEK293T and C666-1 cells), and then slowly decreased, possibly owing to the degradation of the polymers. In all the cell lines, it was observed that 25 %–40 % of the fluorescence intensity was reduced in the GSH-PBAE group compared to that in the PBAE group at each time point. This result indicated that the GSH hybrid strategy could react with intracellular ROS to further minimize fluctuations in ROS levels, thus improving the oxidative stress induced by PBAE polymers.

In addition, C_4_H_6_O_4_-PBAE-plasmid NPs were used to evaluate the intracellular ROS levels because succinic acid is a dicarboxylic acid with the same number of carboxyl groups as GSH. The intracellular fluorescence intensity of the C_4_H_6_O_4_-PBAE group was similar to that of the PBAE group at different time points (1 h, 2 h, 4 h and 8 h), indicating that the reduction of intracellular ROS by GSH-PBAE-plasmid NPs was not mediated by reducing exposure to positively charged protonated ammonia in PBAE (Figure S4).

### GSH hybrid PBAE-NPs reduced disturbance of gene expression in cells

To evaluate the cellular response after gene delivery by cationic polymer NPs, we prepared PEI-GFP5K, PBAE-GFP5K, and GSH-PBAE-GFP5K NPs and delivered them to HEK293T cells. Following 36 h of incubation, we performed RNA-seq to analyze gene expression alterations in contrast to blank group. As a conventional delivery polymer, PEI induced 1,841 up-regulated genes (log2-fold change >1) and 2,489 down-regulated genes (log2-fold change < −1) (Fig. 5c). Similarly, 1,791 upregulated and 3,161 downregulated genes were found in cells delivered by the PBAE-GFP5K NPs, which was close to PEI groups in correlations between the global gene expression (Pearson’s R = 0.99). However, the number of genes altered by the GSH-PBAE-plasmid NPs was remarkably reduced to 789 (373 upregulated and 416 downregulated genes), suggesting reduced disturbance of the GSH-PBAE-GFP5K NPs, which supported the higher similarity between GSH-PBAE-treated cells and blank cells (Pearson’s R = 0.98) (Fig. 5f), suggesting that the GSH hybrid PBAE system can serve as a cell-friendly delivery strategy.

To explore the possible molecular mechanisms that contribute to the different cellular responses, we performed Gene Ontology (GO) enrichment analysis with genes that were altered only in PEI- and PBAE-plasmid NPs delivered cells. It is found that the genes upregulated only in the PEI and PBAE groups were involved in the cellular oxidation response (Figure S5 and Table S3). For example, in the heat map of cellular oxidative stress-response gene expression, 46 genes with high expression were observed in PEI- and PBAE-delivered cells, which demonstrated low expression in blank group. However, after delivery of GSH-PBAE NPs, the expression of these genes recovered (Fig. 5d). A similar tendency appeared in the genes expressing positive and negative regulation of cell growth (Fig. 5e). Overall, the GSH hybrid strategy can reduce the disturbance in gene expression induced by cationic gene delivery system and thus improved biocompatibility.

### In vitro biological evaluation of GSH-PBAE NPs in HEK 293 T cells

The effects of GSH-PBAE and PBAE-plasmid NPs on the cell cycle of HEK 293 T cells. The effects of different NPs on the cell cycle phases were studied (Fig. 5g), and the G1, S, and G2 phases of the blank cells accounted for 43.03 %, 46.09 %, and 5.81 %, respectively. Compared to the blank cells, PEI and PBAE-plasmid NPs induced fewer G phases (30.95 % and 34.97 %, respectively), more S phases (52.24 % and 53.11 %, respectively), and more G2 phases (16.27 % and 9.06 %, respectively). In GSH-PBAE-plasmid NP cells, the G1 phase was 36.06 % and the S phase was 54.31 %, and the G2 phase (5.60 %) was nearly equal to that of the blank cells. The GSH-PBAE NPs had the smallest impact on the cell cycle, which is beneficial for the transcription, translation, and expression of cells. According to the hemolysis assay (detailed in Supplementary Information) (Fig. 5h), for PEI NPs, significant hemolysis (>5%) occurred at only 1.5 μg/mL, whereas for both PBAE cationic polymer NPs, hemolysis occurred at 20 μg/mL and above. However, the GSH-PBAE NPs had a considerably lower hemolysis rate (<5.78 %) than the PBAE NPs (6.95 %) even at 35 μg (PBAE) /ml, indicating that the damage to the cell membrane by GSH-PBAE NPs was relatively mild.

MTT assays were used to further evaluate the damage caused by different cationic polymer-plasmid NPs in HEK 293 T cells (Fig. 5i). For PBAE-GFP5K and GSH-PBAE-GFP5K NPs, the cell vitality was all higher than 80 % at concentrations below 60 μg/ml. However, GSH-PBAE-GFP5K NPs could maintain almost 60 % vitality even at considerably higher concentrations (75–150 μg/ml). In particular, its cell vitality (approximately 60 %) was six times that of non-hybrid PBAE-plasmid NPs (approximately 10 %) at the concentration of 150 μg/ml. In PEI-GFP5K NPs treated group, cell vitality was above 80 % only at concentrations below 4.8 μg/mL, suggesting PEI polymers had greater influence on cell proliferation compared to PBAE, and the GSH hybrid strategy could indeed reduce the impact of PBAE on cell proliferation.

In summary, the GSH hybrid strategy could reduce the cytotoxicity of PBAE polymers by exerting less influence on cell proliferation and the cell cycle, thereby maintaining the normal physiological processes in cells, and may be beneficial for transfection and gene editing of plasmids.

### GSH-PBAE-plasmid NPs improved gene editing efficiency *in vitro*

We chose two target genes (*KAT6A* and *MUC2*) in HEK 293 T cells and one target gene (LMP1) in 293L cells to conduct gene editing efficiency experiments with three types of NPs (PEI, PBAE, and GSH-PBAE). The KAT6A-sg1 plasmids targeting gene *KAT6A* were delivered in HEK 293 T cells, and the *KAT6A* gene Sanger sequences of PCR products demonstrated that only the PBAE and GSH-PBAE groups exhibited obvious “editing peaks” from the 2 to 3 bases before the PAM sequence (AGG), showing that the CRISPR-Cas9 system exerted gene cleavage. Meanwhile, *KAT6A* gene editing efficiency was assessed using the TIDE web tool: PEI group (0.6 %)< PBAE group (7.4 %)< GSH-PBAE group (10.7 %) (Fig. 3h), indicating that the GSH-PBAE-plasmid NPs had a much stronger ability to deliver genes than the non-hybrid PBAE-plasmid NPs.

For the target gene *MUC2* in HEK 293 T cells, T7E1 mismatch detection assay (Fig. 3i) showed that MUC2 plasmid (MUC2P) was successfully delivered by cationic polymer NPs, and the brightness of the bands was as follows: GSH-PBAE group > PBAE group > PEI group, with gene editing efficiencies of 9.2 %, 7.4 %, and 6 %, respectively (Figure S6). In addition, the LMP-g4 and ODN were co-delivered to 293-L cells (Fig. 3j). The PCR products of the primer 1 (LMP-g4-F1+R1) bands in different groups proved the existence of the LMP1 gene. The PCR products of the primer 2 (LMP-g4-F1+ODNR) and primer 3 (LMP-g4-R1+ODNR) bands representing the ODN were successfully inserted to the LMP1 gene. A clear band of primer 2-PCR product only showed up in the GSH-PBAE group, once again proved that the GSH hybrid PBAE strategy could improve gene editing efficiency *in vitro*.

### Biodistribution of GSH-PBAE-plasmid NPs in mice

LMP1 of EBV is a viral oncogene with the potential to antagonize apoptosis and senescence, as well as promote cell survival and proliferation [33]. Nasopharyngeal carcinoma (NPC) is a squamous carcinoma, and recent research indicates that EBV in NPC cells exhibits a type II latency infection that expresses viral membrane proteins, such as LMP1, and EBV exists in NPC cells in the form of extrachromosomal circular DNA [34]. We explored the application of GSH-PBAE-plasmid NPs in the treatment of EBV infection and constructed a mouse head and neck squamous cell line with LMP1 expression (SCC-7-L cells), similar to the establishment of C666-1 cells (a subcloned human NPC cell line) [35]. Information on SCC-7-L cells is shown in Figure S7.

**Fig. 6:**
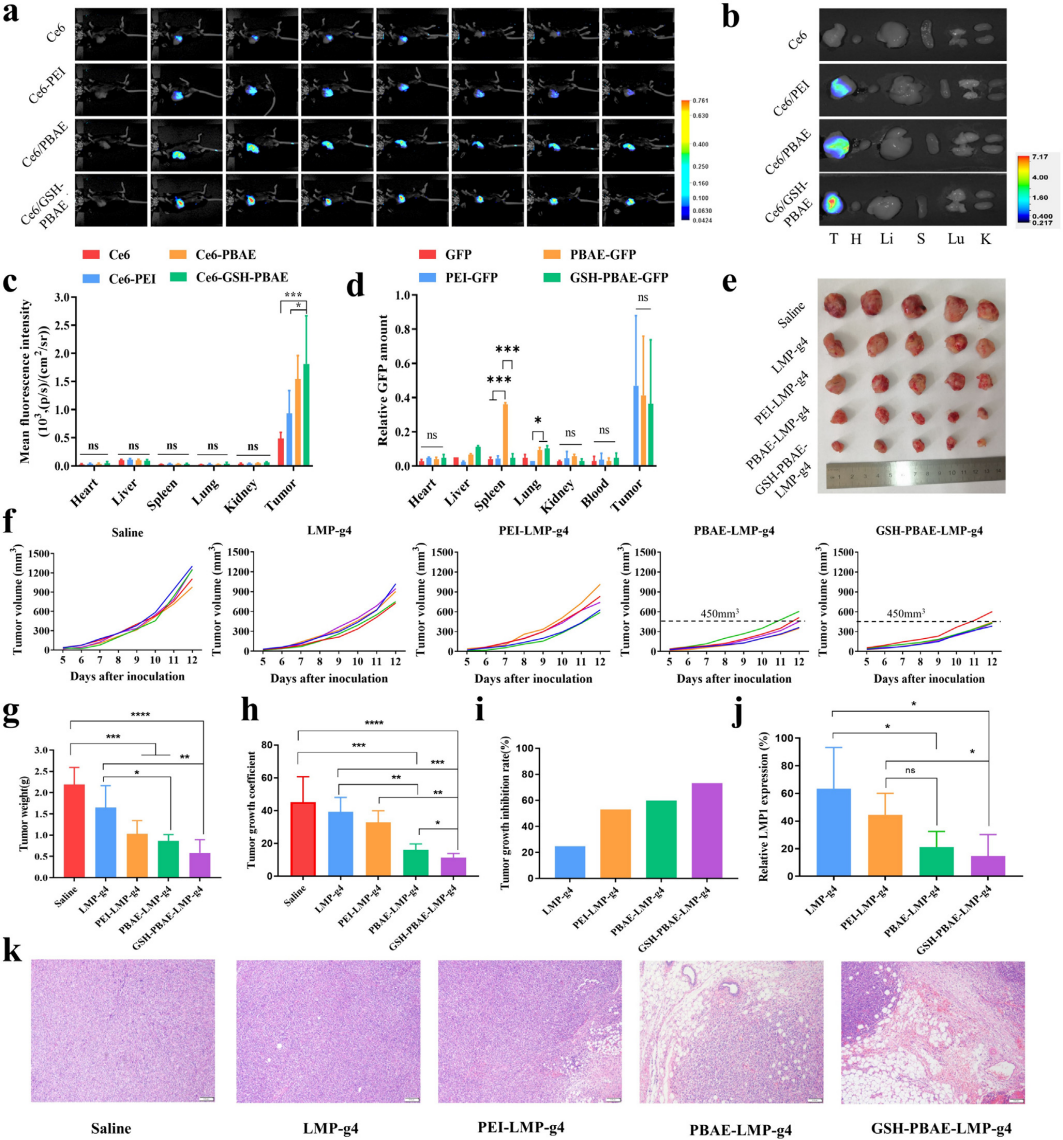
*In vivo* effective evaluation of GSH-PBAE-plasmid NPs in subcutaneous tumors (SCC-7-L) and subcutaneous cell clusters (HEK 293 T). (a) *In vivo* imaging images of different groups at different time intervals in tumor, and (b) the *ex-vitro* imaging of major organs at 24 h. (c) *In vivo* distribution of polymer-LPM1-g4 NPs in SCC-7-L subcutaneous tumors. Mean fluorescence intensity of main organs and tumors after administration for 24 h (n = 3). (d) Relative GFP amount in main organs, blood and tumors (semi-quantitative analysis by PCR and gel electrophoresis) of polymer-GFP NPs in SCC-7-L subcutaneous tumors after peritumoral injection (15 μg plasmids/mouse) for 4 times (control: 1 ng GFP). (e) Tumor images in day 12. (f) Tumors volume change curves after administration of polymer-LPM1-g4 NPs. (g) Tumors weight (n = 5), (h) tumor growth coefficient, (i) tumor growth inhibition rate and (j) quantitative analysis of LMP1 expression in tumors. (k) Representative images of H&E staining of tumor (Scale bar: 100 μm). One-way ANOVA was for statistical analysis, ****p <0.0001, ***p <0.001, **p <0.01, *p <0.05, ns: no significant difference.

The *in vivo* distribution in SCC-7-L subcutaneous tumors showed that the fluorescence intensities of the free Ce6 and Ce6/PEI-LMP-g4 groups decreased gradually over time, particularly in the free Ce6 group, which significantly decreased at 8 h. The Ce6/PBAE-LMP-g4 and Ce6/GSH-PBAE-LMP-g4 NPs still showed strong fluorescence for up to 24 h, indicating the strong retention ability of Ce6/GSH-PBAE NPs (Fig. 6a), which was beneficial to treatment. The tendency of 24-h *ex-vitro* fluorescence intensity (Ce6/GSH-PBAE-LMP-g4 > Ce6/PBAE-LMP-g 4 > Ce6/PEI-LMP-g 4 > Ce6) was consistent with the *in vivo* distribution (Fig. 6b). The fluorescence intensity of NPs in tumor tissues was strong and was extremely low in other organs (Fig. 6c). To further reveal the biodistribution of the NPs and plasmids, the amount of plasmids delivered to the tumors and organs was measured by PCR (Fig. 6d and Fig. S8) using GFP as the model. After 4 times of peritumoral injection, perhaps due to the diffusion of NPs around the tumor, a small amount of GFP can be detected in the blood and other organs. For PBAE-GFP NPs and GSH-PBAE-GFP NPs, their enrichment in liver and lung was stronger than in other organs (no significant difference in GFP content in blood, heart, and kidney), suggesting that macrophages may play a role in capturing the NPs. It is worth noting that there was a higher spleen accumulation for PBAE-GFP NPs, which may be related to the immune response inducing capability of PBAE according to our previous research [36]. Interestingly, GSH hybridization can weaken this effect. In the spleen, GSH-PBAE-GFP NPs is similar to PEI-GFP NPs and GFP. Future studies are still needed to reveal the causes and mechanisms of NPs’ distribution. These results also suggested that even with local administration such as peritumoral injection, the systemic distribution still needed to be considered, which put higher demands on the safety evaluation of gene therapy.

Furthermore, we established a mouse subcutaneous HEK293T cell cluster model to determine the tissue distribution of different NPs (Figure S9). The results for the HEK293T cell clusters were consistent with those for SCC-7-L tumors. Ce6/GSH-PBAE-LMP-g4 showed longest retention in cell cluster. We further used reporter gene (luciferase plasmids) to evaluate the *in vivo* gene transfection of the NPs (Figure S10a). The fluorescence intensity of GSH-PBAE NPs group (3 and 4) were all much stronger than that of PBAE group (1 and 2) could directly demonstrate that the GSH hybrid improved the gene delivery ability of PBAE *in vivo*.

### Efficiency evaluation of GSH-PBAE-plasmid NPs in mice

Therapeutic effect of different cation polymer-plasmids NPs was assessed on SCC-7-L subcutaneous tumors model (Figure S11). Photographs of the tumors were taken on the 12th day (Fig. 6e) and their volume, size, and weight were measured. As illustrated in Fig. 6f, the tumor volumes in control group (Saline) of all mice were greater than 900 mm^3^ on day 12. As expected, the free plasmid LMP-g4 led to only a slight decrease in tumor growth, with tumor volumes between 600 mm^3^ and 1200 mm^3^. The antitumor efficacy of the PEI-plasmid NPs was slightly better than that of the free plasmid, and tumor volumes less than 600 mm^3^ were observed in only two mice. Interestingly, we found that two tumors were ≤450 mm^3^ in the PBAE group whereas four were observed in the GSH-PBAE group. The tumor growth coefficient showed that in GSH-PBAE-LMP-g4, the tumor volume increased for only 11 times while for PBAE-LMP-g4 and PEI-LMP-g4, the values were 16 and 33, respectively. More importantly, the tumor weights in the GSH-PBAE group (0.6 g) were 30 % lower than those in the PBAE group (0.87 g) (Fig. 6g), and the tumor growth inhibition rate values for them were 73 % and 60 %, respectively, indicating that GSH hybrid could improve the anti-tumor efficiency of the NPs. For LMP-g4 plasmids alone, owing to the negative charge and low stability, both the tumor growth coefficient and tumor growth inhibition rate were close to saline group without significant difference (Fig. 6h, i). Only with the help of cationic polymers, can they penetrate in tumor tissue, entering the cells and then transfect into the CRISPR/Cas9 system. When replacing the plasmid with GFP (without gene editing capability), it can be seen that there is no significant difference in tumor growth and weight among the four groups (Figure S12), which also indicated that the NPs themselves have no inhibitory effect on tumor growth. Quantitative analysis of LMP1 expression in tumors revealed that the expression of LMP1 after treatment with GSH-PBAE-LMP-g4 NPs (approximately 15 %) was lower than that in the PBAE/LMP-g4 group (21 %), and much lower than that in the PEI-LMP-g4 group (44 %) (Fig. 6j). Sanger sequences results (Figure S13) demonstrated that in GSH-PBAE groups, the *LMP1* gene editing efficiency was 6.5 % 24 h after a single administration as assessed by the TIDE web tool. For PBAE-LMP-g4, the value was 4.4 % and in PEI group it’s only 0.8 %. Although the total efficiency is not high, it still exhibits good tumor growth inhibition, which may be related to the sensitivity of cells containing *LMP1* to gene editing. In our previous research, we also found that even if the efficiency is below 10 %, the CRISPR/Cas9 system showed good growth inhibition ability on *LMP1* positive C666-1 cells [37]. However, further research is still necessary to explain the relationship between gene editing and cell growth inhibition of these cells. The H&E-stained sections of the tumors showed that the tumor tissue cells in the GSH-PBAE-LMP-g4 group had condensed nuclei, significantly enlarged intercellular spaces, and loose connections between cells, in contrast to the intact cellular morphology of the tumor tissues in the saline and LMP-g4 groups (Fig. 6k), suggesting that the GSH-PBAE-LMP-g4 NPs exhibited strong tumor cytotoxicity and tumor growth inhibition.

*In vivo* gene editing testing in HEK 293 T subcutaneous cell clusters bearing mice were also investigated on *KAT6A via* OND-PCR (detailed in Supplementary Information). As shown in Figure S10b, bands appeared in both the two groups treated with PBAE-KAT6A-sg1 NPs and GSH-PBAE-KAT6A-sg1 NPs, demonstrating that the ODN was successfully inserted into the disruption site created by the cas9 nuclease.

### Safety evaluation of GSH-PBAE-plasmid NPs in mice

**Fig. 7:**
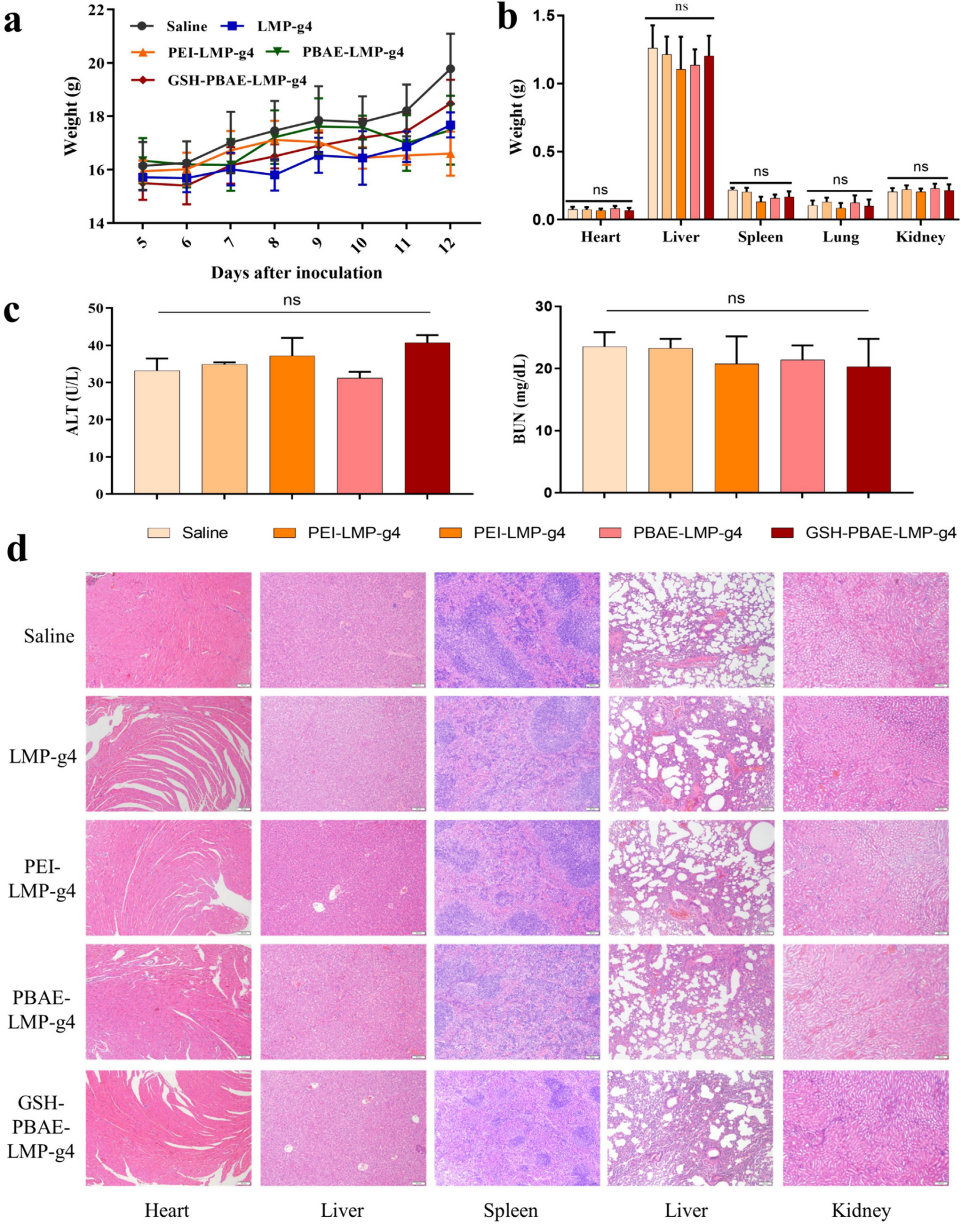
Safety evaluation after administration of different NPs. (a) Body weight of mice (n = 5). (b) Weight of major organs (n = 5). (c) ALT (n = 2) and (d) BUN (n = 2). (e) Representative images of H&E staining of heart, liver, spleen, lung and kidney in each group (Scale bar: 100 μm). One-way ANOVA was for statistical analysis, ns: no significant difference.

We also evaluated the *in vivo* safety of the polymer-plasmids NPs. The weights of the mice in each group fluctuated steadily during the administration period (days 5 to 12), and none of the mice showed significant weight loss (Fig. 7a). The organ weights of all mice were similar (Fig. 7b). The blood biochemical results showed that both ALT and BUN were all within the normal reference range (ALT: 10.06–96.47 U/L, BUN: 10.81–34.74 mg/dL), indicating that none of the NPs caused liver or kidney damage (Fig. 7c). H&E staining images of different organs showed that the myocardial cell structure in each group was intact, and the structures of the hepatic sinusoids, liver lobules, splenic sinusoids, alveolar capillaries, and glomeruli were similar to those in the saline-treated control group, with no cell swelling or lymphocyte infiltration and with a complete cellular structure, indicating that these polymer-plasmids NPs had no significant tissue toxicity (Fig. 7d).

Furthermore, according to the *in vitro* hemolysis assay, we performed a preliminary *in vivo* exposure safety evaluation of the three types of polymer-plasmid NPs (see Supplementary Information). The mice injected with PEI-plasmid NPs at >2.8 mg PEI/kg begun to die in 30 min, whereas the value for mice injected GSH-PBAE-plasmid NPs was 171.4 mg PBAE /kg, which was also higher than that of PBAE-plasmid NPs (85.7 mg PBAE/kg). These results suggest the *in vivo* safety of PBAE polymers was enhanced after GSH hybrid strategy.

## Discussion

In this study, the GSH hybrid PBAE-plasmid NPs possessed a good stability in size and a higher plasmid encapsulation capacity, indicating that GSH did not affect the process of forming NPs between PBAE and plasmid although the ζ-potential in the GSH-PBAE-plasmid NPs slightly decreased about 7 mv. Because it did not change the cell uptake mechanism (mainly membrane fusion) and plasmids encapsulation efficiency of the PBAE polymer, the transfection efficiency and gene editing efficiency enhanced by GSH hybrid system may be due to the difference in downstream events after cell uptake of the NPs. It is demonstrated that GSH-PBAE-plasmid NPs reduced ROS induced by cationic polymer PBAE, protected cells from oxidative stress, and thus minimized gene expression disturbance. In vitro biological evaluation further proved that the GSH hybrid NPs had less influence on cell proliferation and cell cycle regulation. In EBV-infected mouse tumor model, although the difference in the retention of plasmids in tumors between GSH-PBAE-plasmid NPs and PBAE-plasmid NPs is not significant, gene editing efficiency of GSH-PBAE-LMP-g4 NPs was improved compared to PBAE-LMP-g4, perhaps due to the modulation of intracellular oxidative stress and reduced disturbance of host cell gene expression. As a result, GSH-PBAE-LMP-g4 NPs exhibited good growth inhibition of tumors with much lower expression of LMP1 compared to PBAE-LMP-g4 NPs and PEI −LMP-g4 NPs.

As a cationic gene delivery system, although GSH hybrid have greatly improve the bio-safety of PBAE-plasmid system *via* intravenous injection, a better way to use such gene-editing system to achieve gene therapy is still local administration, such as human papilloma virus (HPV)/EBV infection, cervical cancer and nasopharyngeal carcinoma, which can be administered directly through vagina and nasal. Further investigation is needed to comprehensively evaluate the *in vivo* behavior and expand the application prospects of this system.

## Conclusions

Simply, we reported a simple and effective gene delivery system using a GSH hybrid strategy. We demonstrated that the GSH-PBAE-plasmid NPs is a good gene delivery vector in terms of safety and gene delivery *in vitro* and *in vivo*. It has the potential to be expanded to the field of gene editing technology based on CRISPR-Cas9 for various gene treatments. The development of a simple and effective strategy is expected to promote clinical applications of non-viral vector gene delivery.

## Compliance with Ethics requirements

All Institutional and National Guidelines for the care and use of animals (fisheries) were followed; and the Institutional Animal Care and Use Committee (IACUC) number was HLK-20231110-001.

## Declaration of Competing Interest

The authors declare that they have no known competing financial interests or personal relationships that could have appeared to influence the work reported in this paper.
