# Supplementary material for: Glutathione hybrid poly (beta-amino ester)-plasmid nanoparticles for enhancing gene delivery and biosafety
Source: J Adv Res. 2024 Aug 2;73:697–711. doi: 10.1016/j.jare.2024.07.038 (PMC12225941; doi:10.1016/j.jare.2024.07.038)
Supplement: Supplementary Data 1 [file mmc1.doc]

| **Glutathione hybrid poly (beta-amino ester)-plasmid nanoparticles for enhancing gene delivery and biosafety** |
| --- |

**Figure S1 in the Electronic Supplementary Material (ESM)**

**
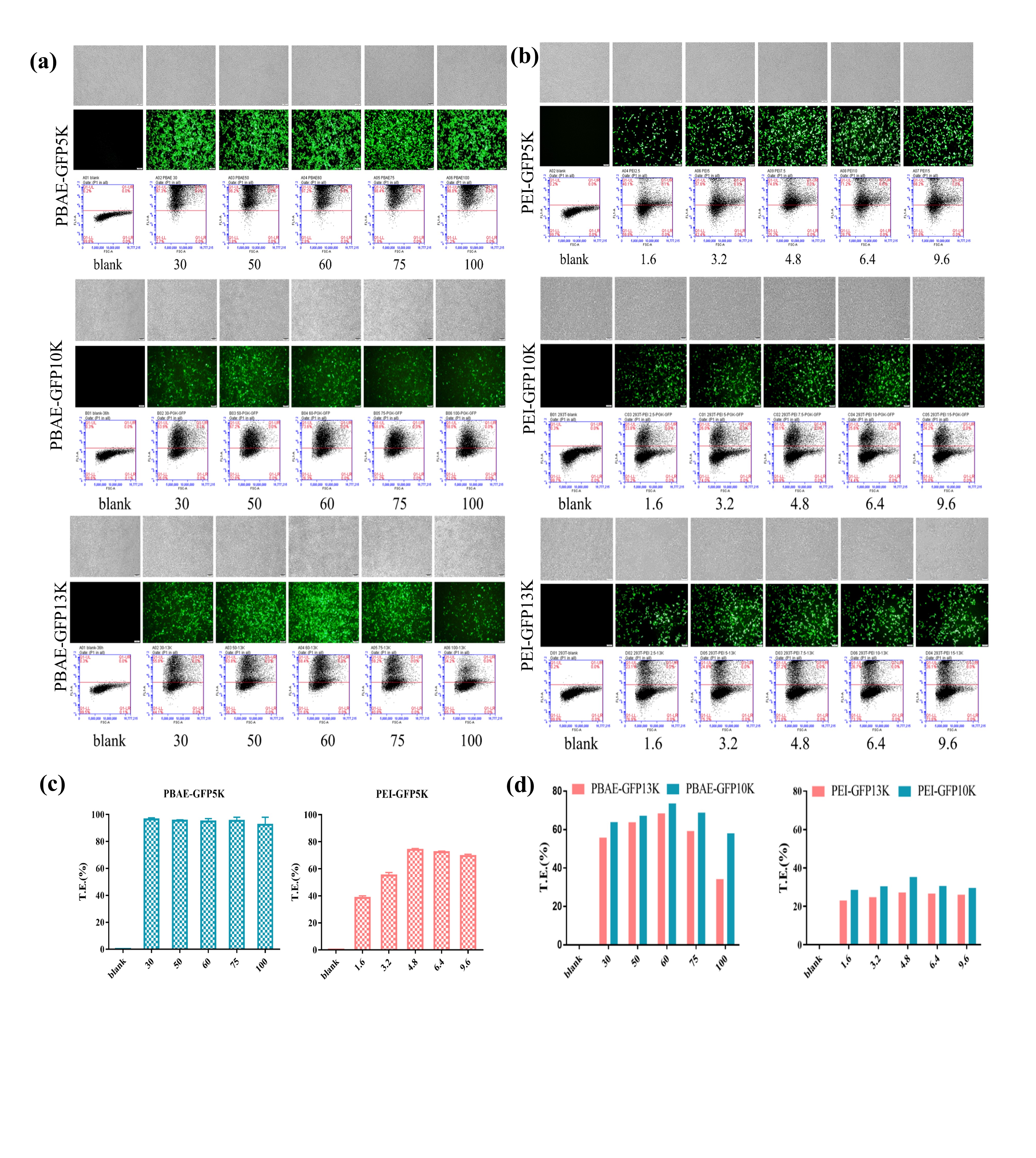
**

**Figure S1.** The T.E. of PBAE-plasmid NPs and PEI-plasmid NPs on HEK 293T cells along at various mass ration using different plasmids. Typic fluorescence microscopy and flow cytometry of (a) PBAE-GFP5K/GFP10K/GFP13K NPs on HEK 293T cells (Scale bar: 100 μm). (b) PEI-GFP5K/GFP10K/GFP13K NPs and Corresponding statistics of transfection efficiency of (c) PBAE-GFP5K NPs, PEI-GFP5K NPs and (d) PBAE-GFP13K/10K NPs and PEI-GFP13K/10K NPs (n = 3).

**Figure S2 in the ESM**


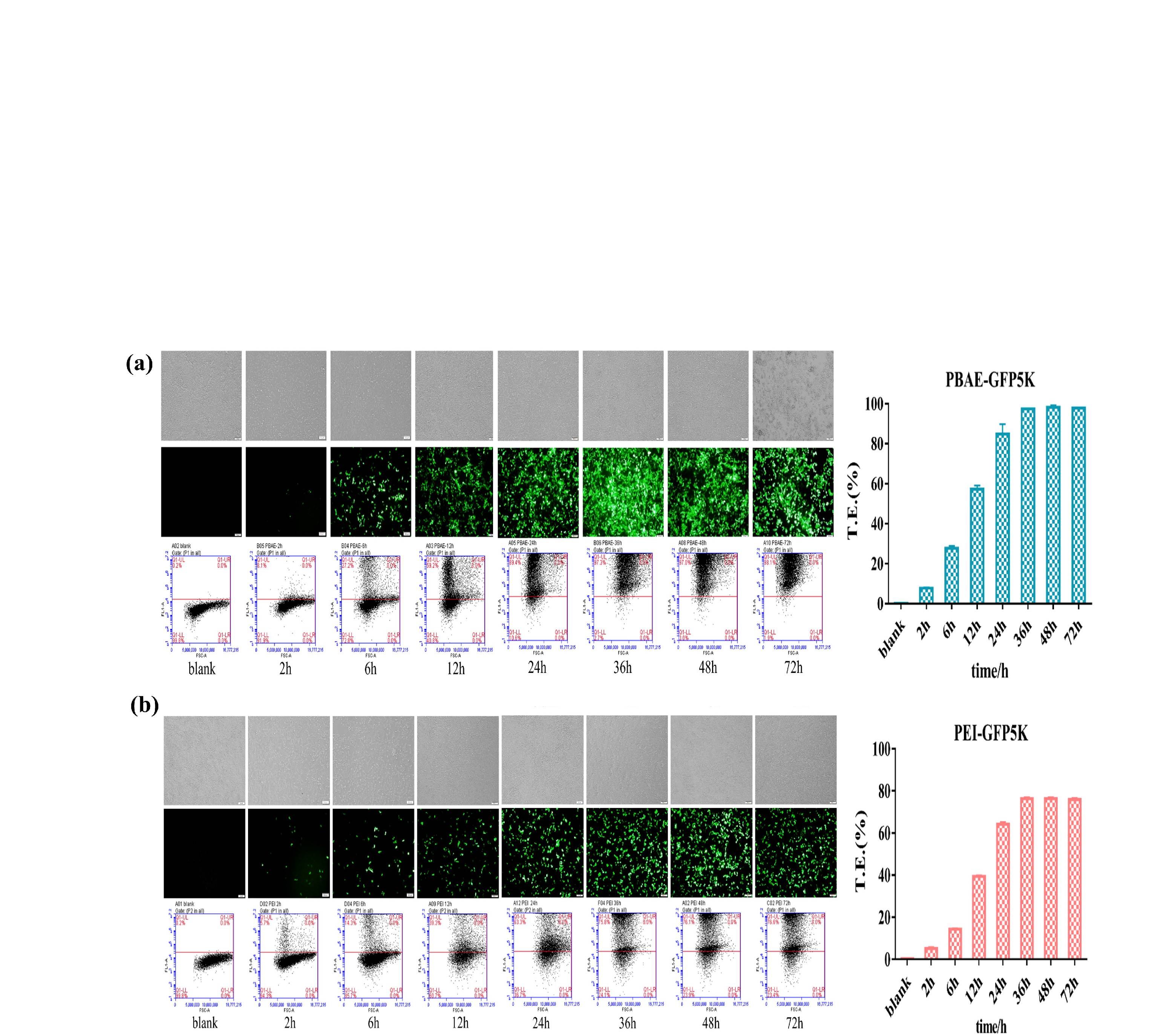


**Figure S2.** Transfection efficiency of PBAE/PEI-GFP5K polyplex NPs on HEK 293T cells for different intervals. Typic fluorescence microscopy and flow cytometry of (a) PBAE-GFP5K NPs and (b)PEI-GFP5K NPs on HEK 293T cells (Scale bar: 100 μm), and Corresponding statistics of transfection efficiency of (c) PBAE-GFP5K NPs and (d) PEI-GFP5K NPs (n = 3).

**Figure S3 in the ESM**


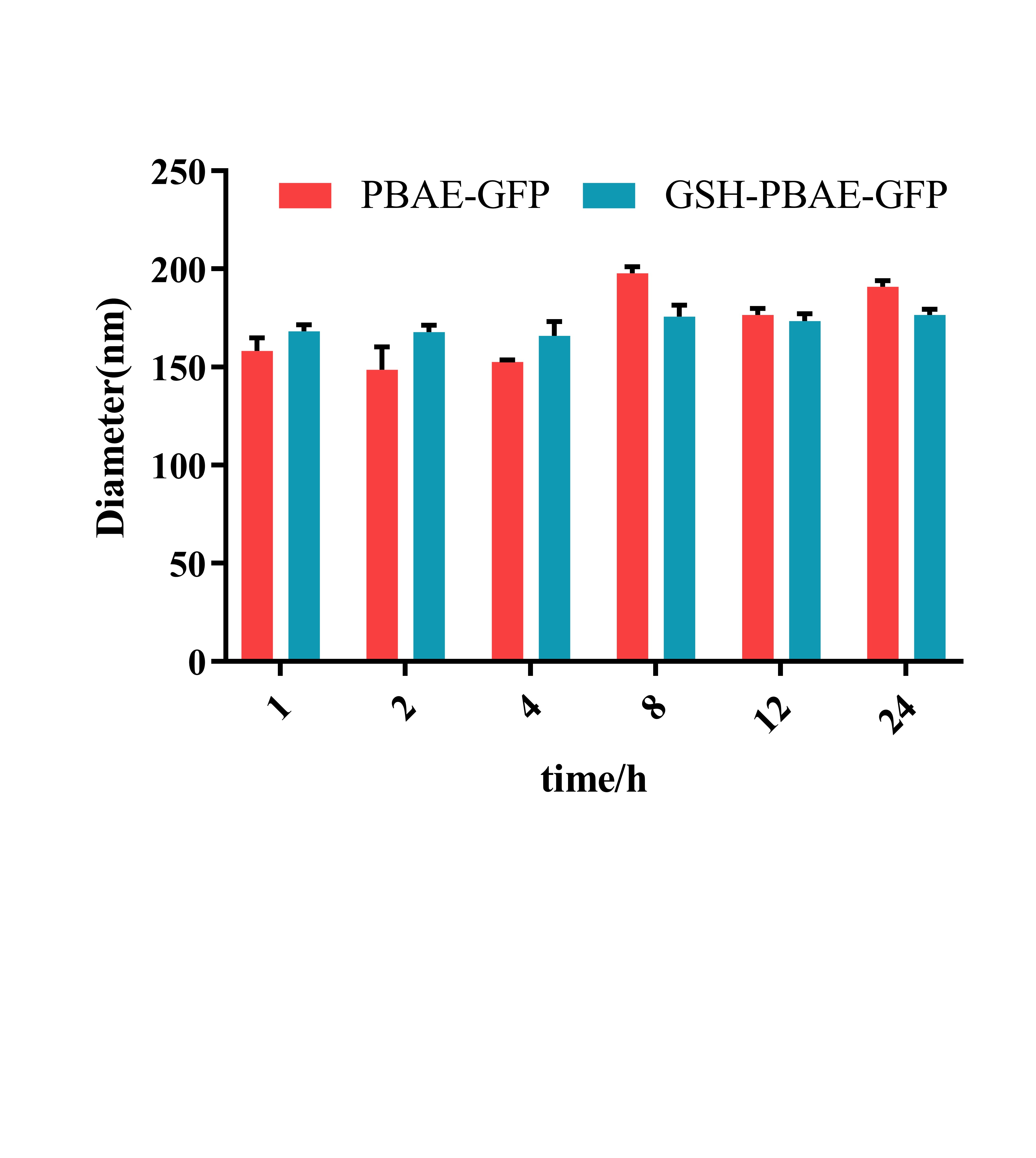


**Figure S3.** Stability of PBAE-GFP5K polyplex NPs and GSH-PBAE-GFP5K polyplex NPs in FBS at room temperature.

**Figure S4 in the ESM**


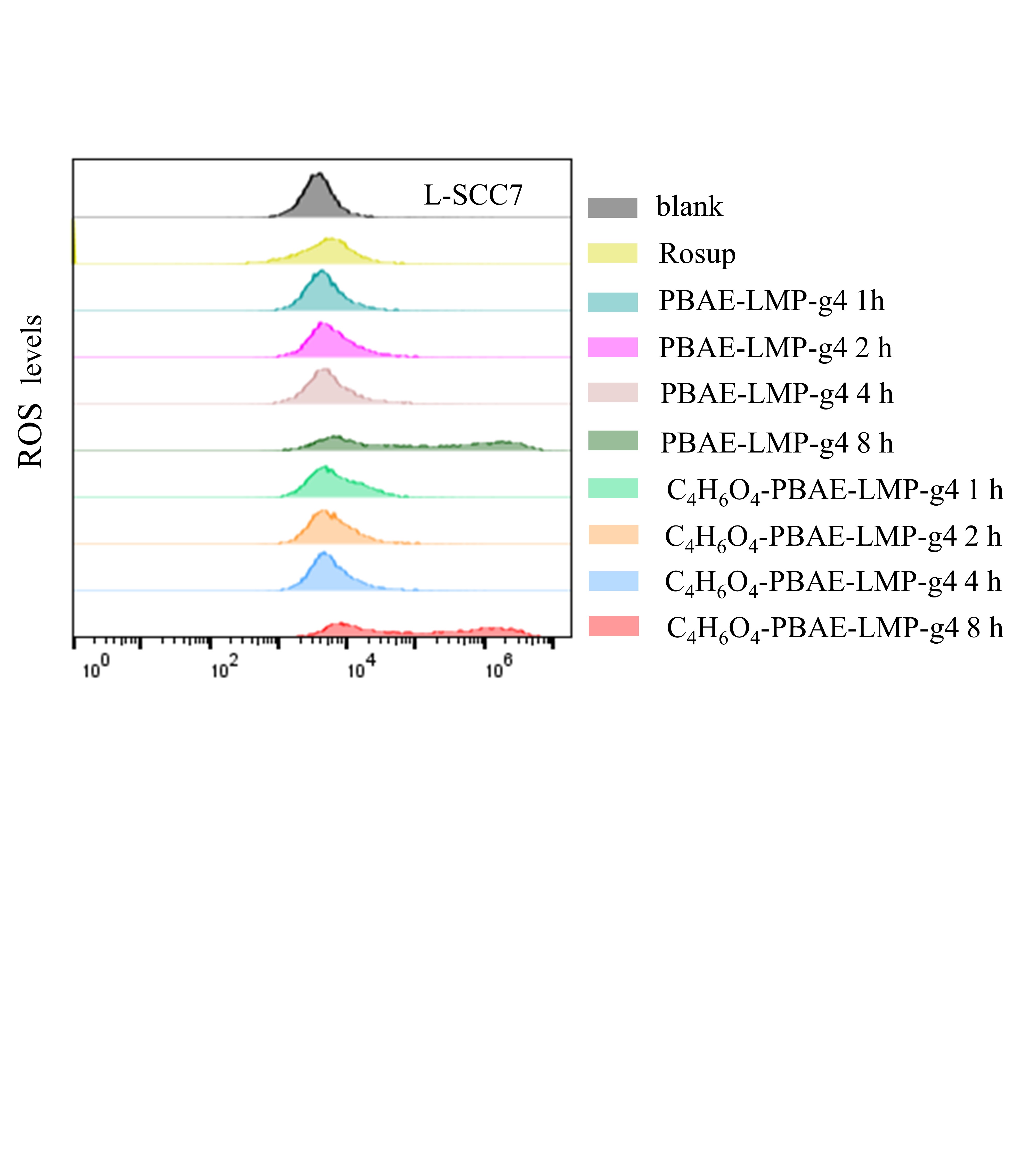


**Figure S4.** Intracellular ROS levels of SCC-7-L incubated with succinic acid (C_4_H_6_O4) hybrid PBAE-LMP-g4 NPs at different time points compared to nonhybrid PBAE-LMP-g4 NPs.

**Figure S5 in the ESM**


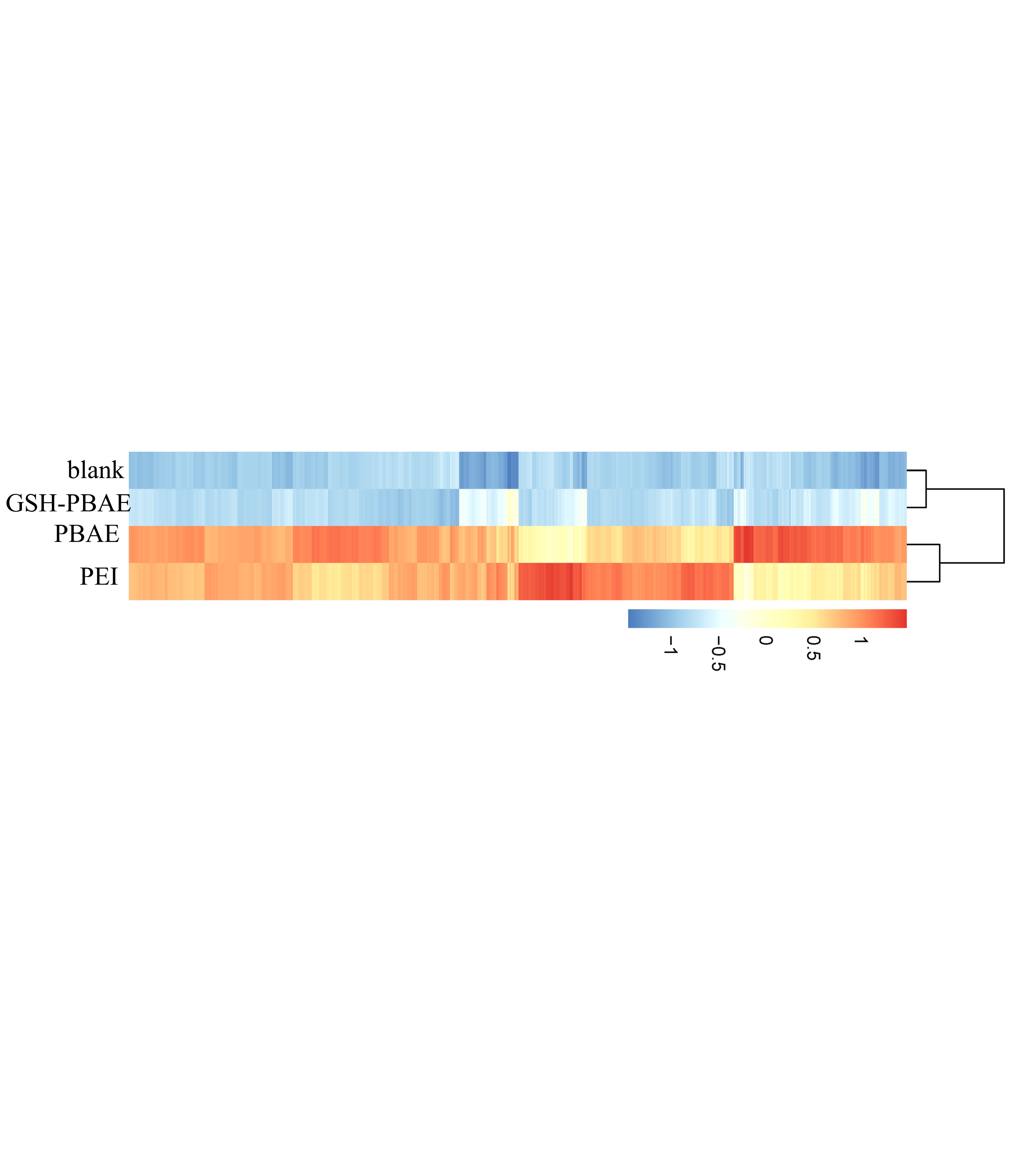


**Figure S5.** The heat map of up-regulated differential genes in PEI and PBAE polymers delivered HRK 293T cells.

**Figure S6 in the ESM**


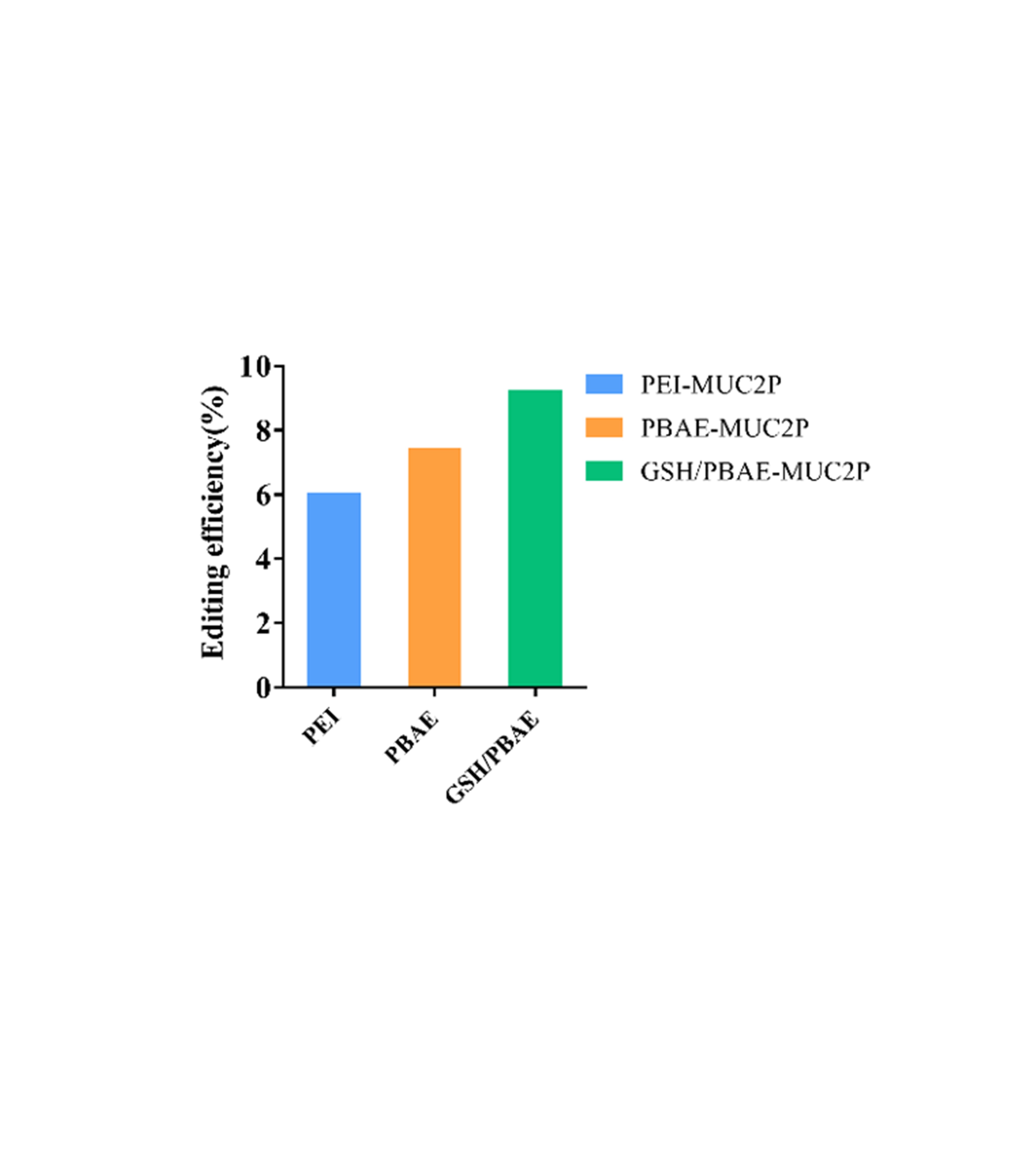


**Figure S6.** The quantitative results of editing efficiency of gene editing of MUC2 gene in HEK 293T cells

**Figure S7 in the ESM**

Here, we provide information on the construction of the SCC-7-L cell lines. SCC-7-L cell DNA was extracted, and PCR was performed using the primer shown in Table S3(LMP1). It was found that SCC-7-L had bands similar to those of the positive control (Figure S7(a)), and we further compared the LMP1 gene sequences in the CCC-6 cell line with those of the constructed SCC-7-L cell line; the results demonstrated that they were completely consistent. Therefore, all the information supported the conclusion that the construction was successful (Figure S7(b)). The construction method of 293-L cells was similar to that of SCC-7-L cells.


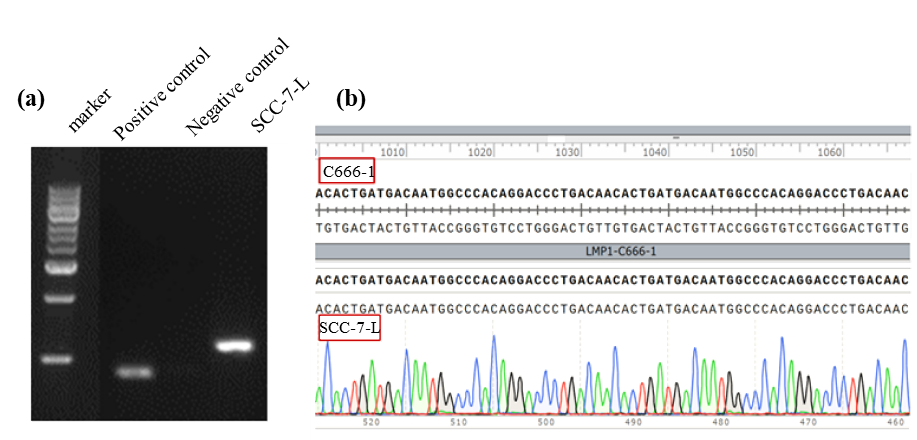


**Figure S7.** The information about SCC-7-L cell line. (a) Agarose gel electrophoresis of PCR products after construction about SCC-7-L cells (positive control was C666-1cells group with LMP1 bond and negative control was the SCC-7 cells without LMP1 bond). (b) the sequences of LMP1 gene in C666-1 cell and in SCC-7-L cell.

**Figure S8 in the ESM**


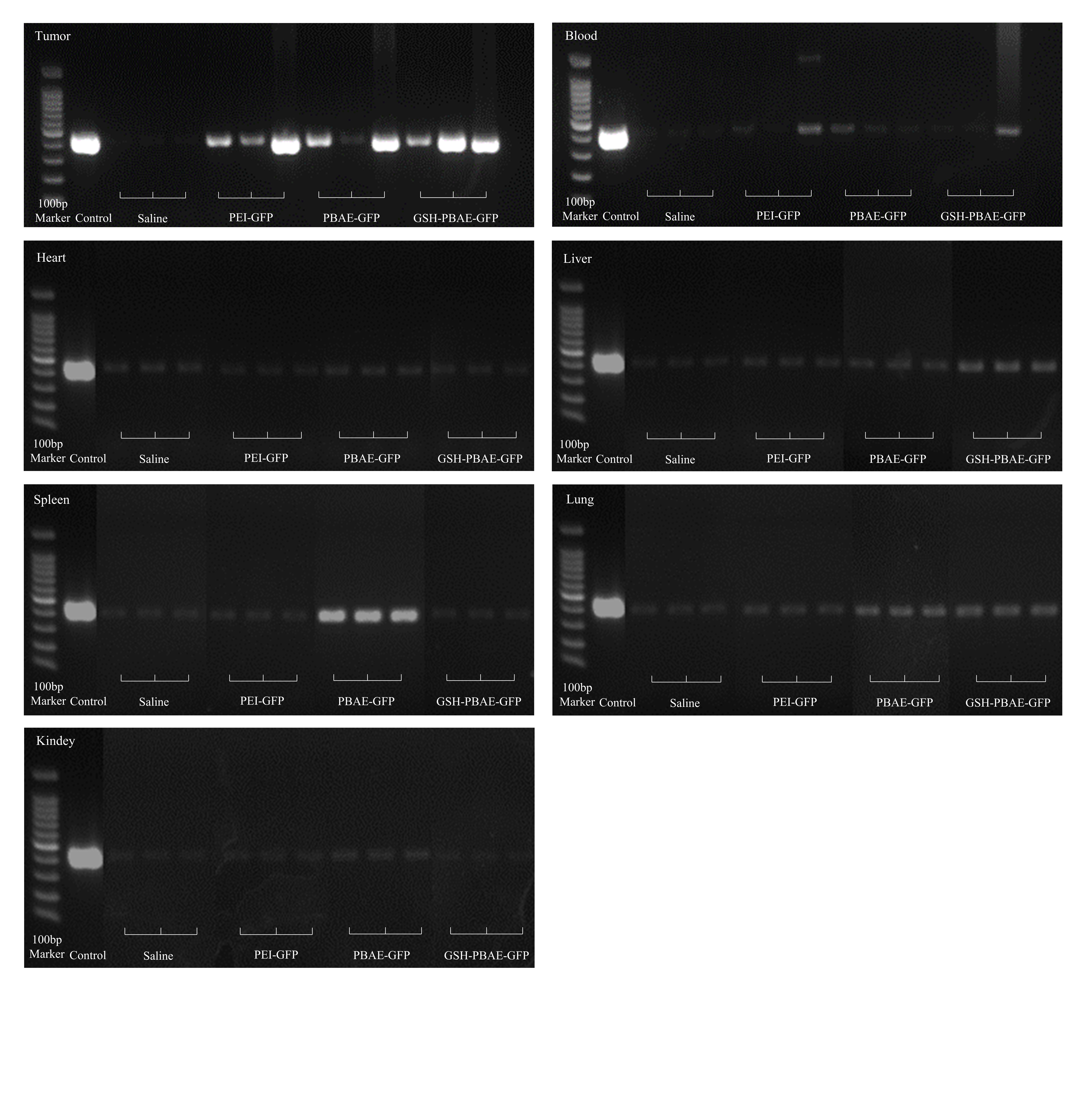


**Figure S8.** Gel electrophoresis results of tumors, blood and major organs (heart, liver, spleen, lung and kidney) after peritumoral injection of GFP5k, PEI-GFP5k NPs, PBAE-GFP5k NPs, and GSH-PBAE-GFP5k NPs (15 μg plasmids/mouse) for 4 times in SCC-7-L subcutaneous tumors. control: 1ng GFP.

**Figure S9 in the ESM**

**Biodistribution of GSH/PBAE-plasmid NPs in Subcutaneous HEK 293T cells clusters of mice**

**Method.** The healthy balb/c nude mice were injected subcutaneously with HEK293T cells in the right axillary skin to form tumor models (50-100 mm^3^). The mice were randomly divided into four groups (three mice per group): free Ce6 group, Ce6/PEI-LMP-g4 NPs group, Ce6/PBAE-LMP-g4 NPs group, and Ce6/GSH-PBAE-LMP-g4 NPs group. The Ce6-PBAE was used for NPs preparation and in vivo tracking. NPs were administered by peritumoral injection. After 0.5 h, 1 h, 2 h, 4 h, 8 h, 12 h, 24 h and 48 h, the mice were narcotized and placed in the mouse in vivo imaging system (700 nm) to observe the accumulation and retention of NPs. Then the mice were sacrificed after 24 h, The tumors and major organs (hearts, livers, spleens, lungs, and kidneys) were collected and placed under mouse in vivo imaging system for observation.

**Results.** The in vivo distribution of each group in the subcutaneous cluster of HEK 293T cells after peritumoral injection is shown in Figure S9. The fluorescence intensities of the cell cluster site in the free Ce6, Ce6/PEI-LMP-g4, and Ce6/PBAE-LMP-g4 groups gradually decreased over time, decreased significantly at 12 h, and almost disappeared at 48 h. In contrast, the fluorescence in the cell cluster site of the Ce6/GSH-PBAE-LMP-g4 group remained strong even at 48 h (Figure S9A), indicating that the Ce6/GSH-PBAE-LMP-g4 NPs remained in the cell cluster site for a long time, contributing to the effective treatment of the plasmids. The in vitro results after 48 h showed that the fluorescence intensity of each group in the cell cluster site was in the order Ce6/GSH-PBAE-LMP-g4 > Ce6/PBAE-LMP-g4 > Ce6/PEI-LMP-g4 > free Ce6, which was consistent with the in vivo distribution results (Figure S9B). Moreover, the fluorescence intensity of each group in the main organs (heart, liver, spleen, lung, and kidney) was quite low and exhibited no significant differences (Figure 6C).


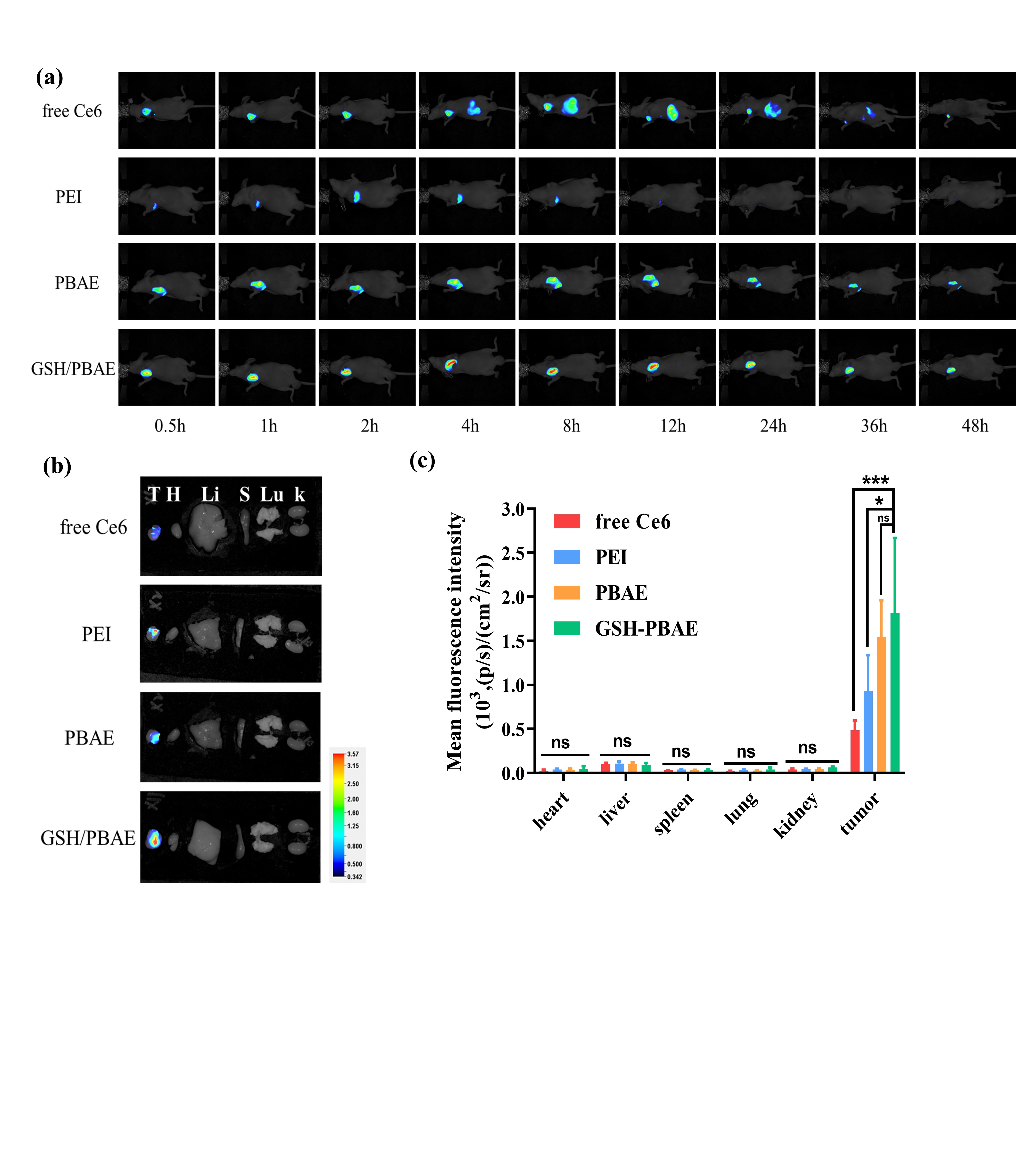


**Figure S9.** *In vivo* distribution of GSH/PBAE-plasmids polyplex NPs in HEK 293T subcutaneous cell clusters. (a) In vivo imaging images at different points. (b) In vitro imaging of major organs at 48 h. (c) Mean fluorescence intensity of hearts (H), livers (Li), spleens (S), lungs (Lu), kidneys (K) and cell clusters (T) after administration for 48 h (n = 3). One-way ANOVA was for statistical analysis, ***p<0.001, *p<0.05, ns: no significant difference.

**Figure S10 in the ESM**

**Method.** The HEK 293T subcutaneous cell clusters bearing mice were established as above (method in Figure S9). The clusters were then injected by 10mg luciferase reporting gene plasmids with PBAE-plasmids ratio of 60:1 for PBAE-plasmid and GSH-PBAE-plasmids group. After 48 h, D-Luciferin potassium salt (10mg/ml) was intraperitoneally injected and observed on an in vivo imaging system (nightowl LB983, Berthold) 10 minutes later.

For *in vivo* gene editing testing, the HEK 293T subcutaneous cell clusters bearing mice were peritumorally injected by PBAE-KAT6A-sg1 NPs and GSH-PBAE-KAT6A-sg1 NPs (10 μg plasmids and 2ug ODN/mouse). After 48 h, the mice were sacrificed and the clusters were collected. The total DNA of the samples was extracted by Genomic DNA Kit (TransGen, EE101-11), amplified by PCR, and detected by gel electrophoresis.


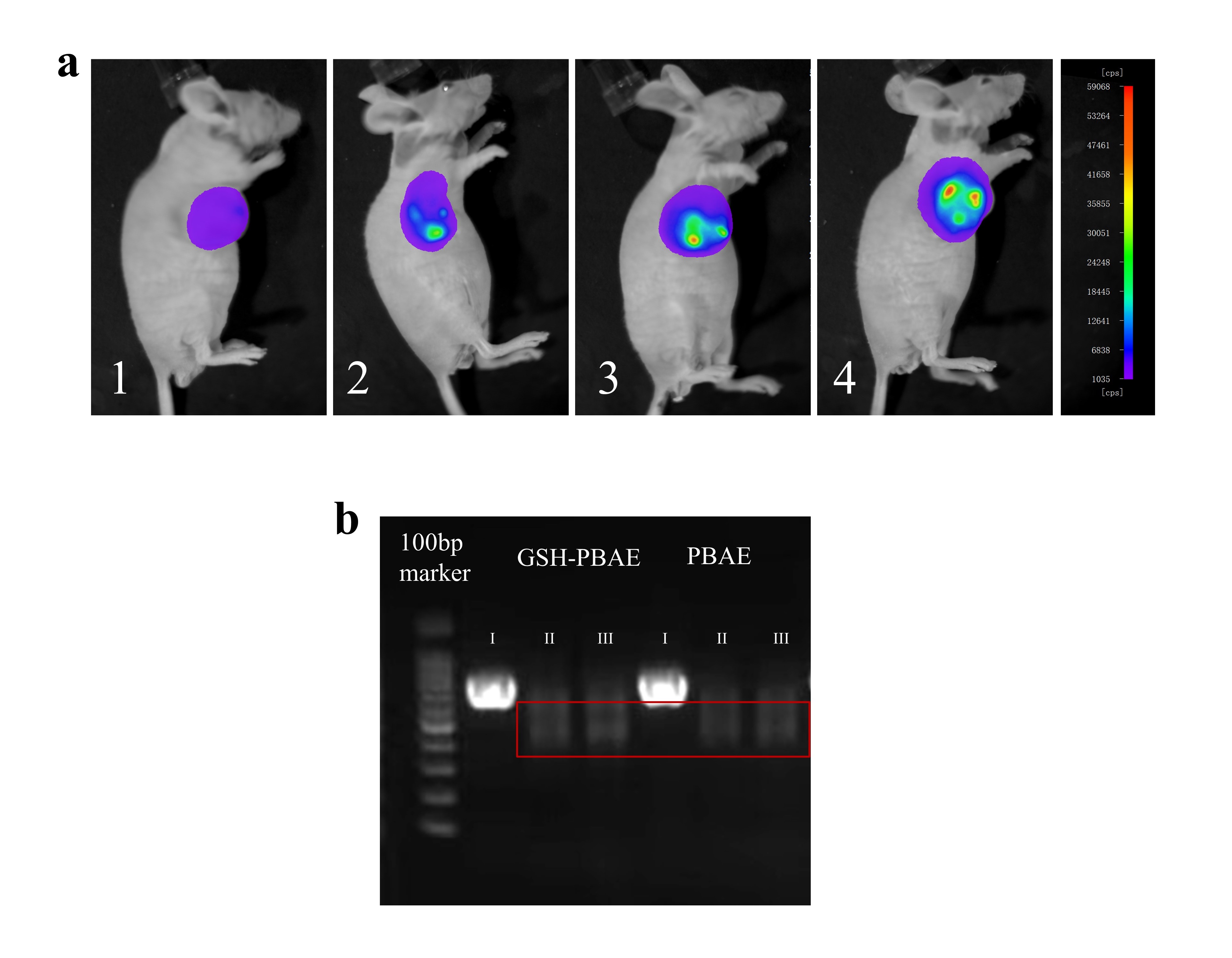


**Figure S10.** *In vivo* transfection evaluation in HEK 293T subcutaneous cell clusters. (a) in *vivo images* of luciferase expression in different groups (1 and 2 were the PBAE group and 3 and 4 were the GSH-PBAE group). (b) Agarose gel electrophoresis results after ODN-PCR (primers: I: KAT6A-F & KAT6A-R, II: KAT6A-F & OND-R, III: ODN-F & KAT6A-R)

**Figure S11 in the ESM**


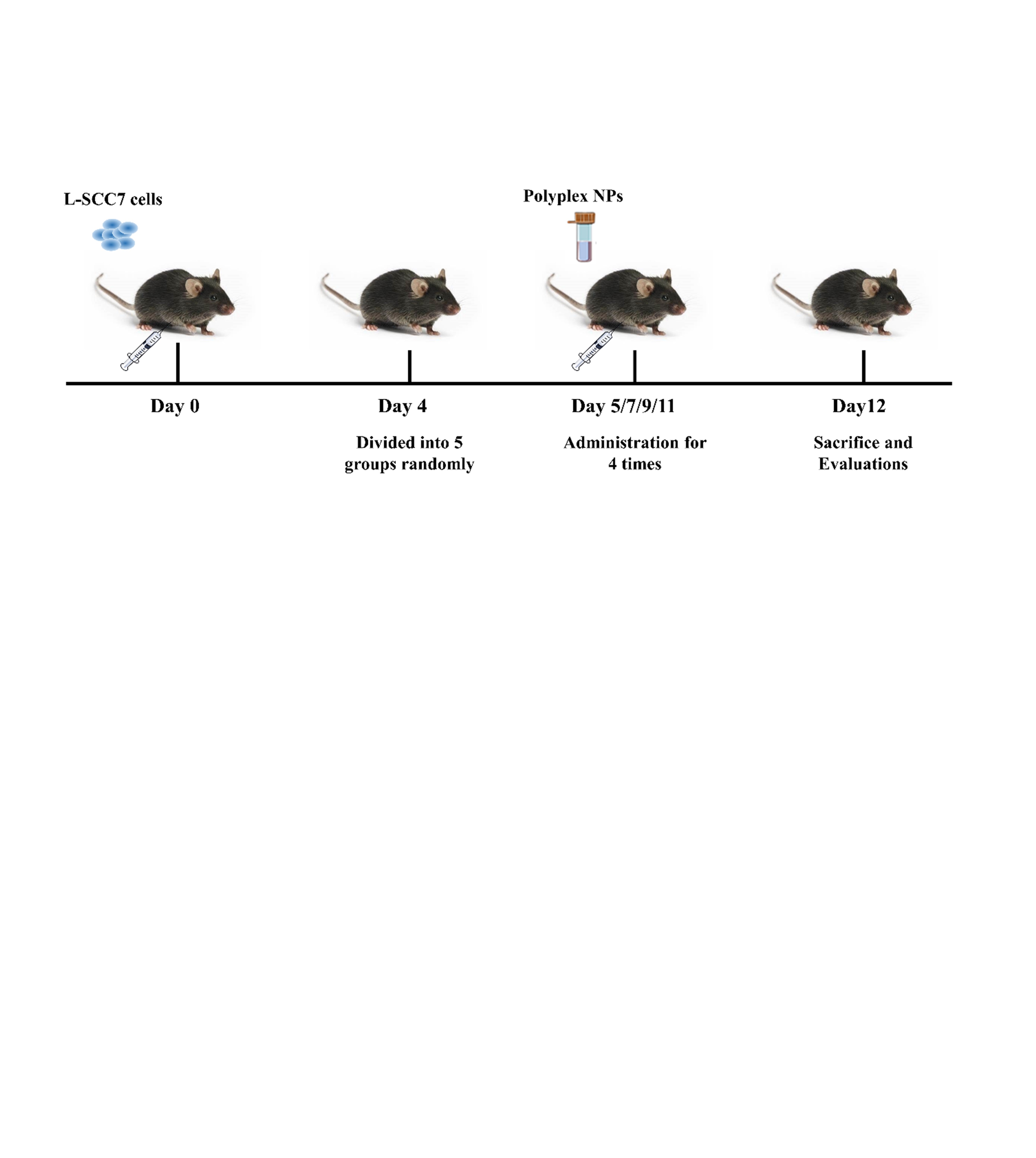


**Figure S1****1.** Schematic illustration of growth inhibition of GSH/PBAE-LMP-g4 polyplex NPs in vivo.

**Figure S12 in the ESM**


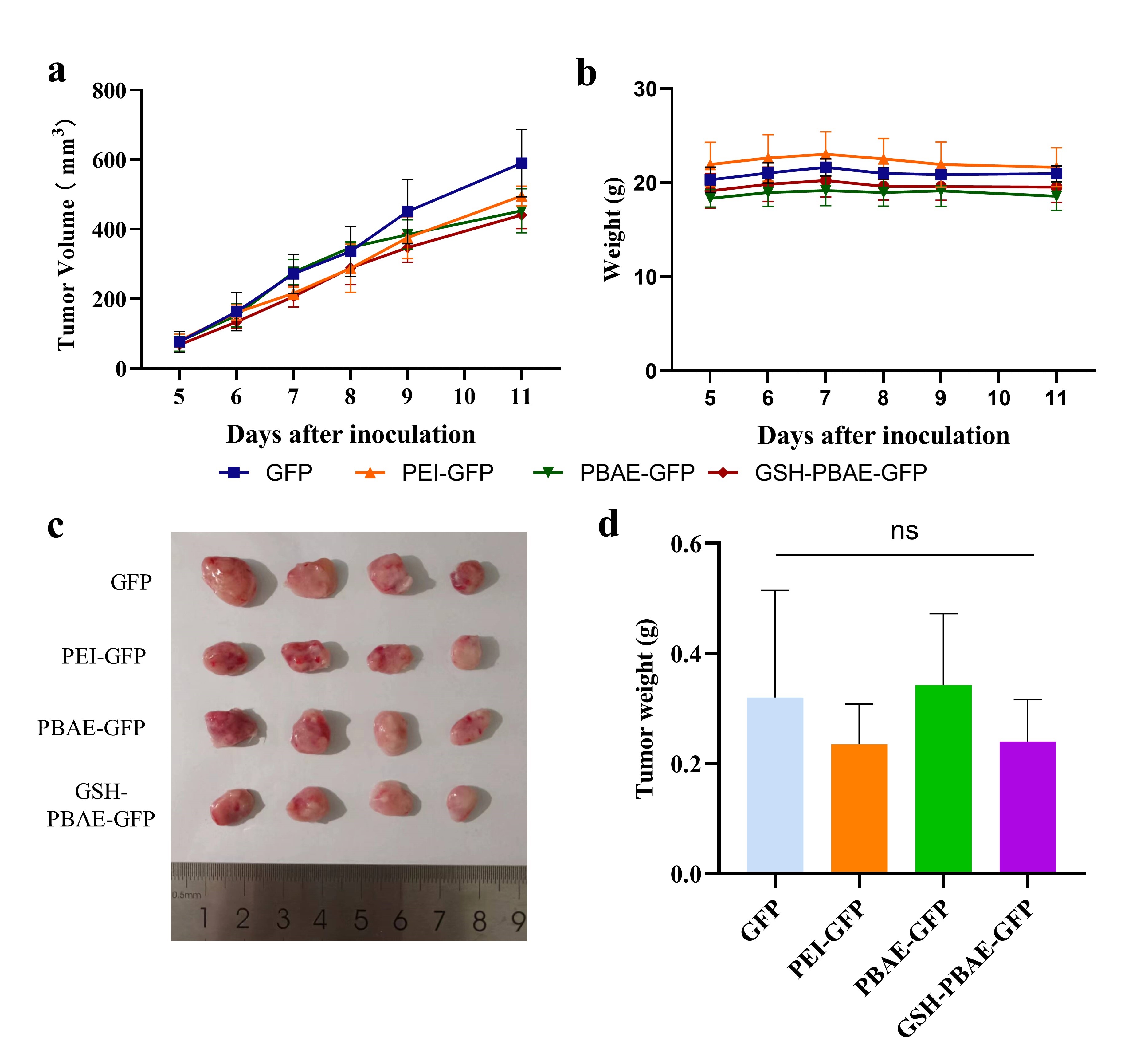


**Figure S12.** *In vivo* SCC-7-L xenograft tumor growth treated by different polymer-GFP NPs. (a) tumor volume, (b) body weight, (c) tumor images, (d) tumor weight. n=4. One-way ANOVA was for statistical analysis, ns: no significant difference.

**Figure S13 in the ESM**

**Figure S13.** The *in vivo* gene editing results of sanger sequences of PCR products (LMP1 gene) after delivering LMP1-g4 plasmids in SCC-7-L tumor bearing mouse model and the total editing efficiency analyzed by TIDE.

**Table S1 in the ESM**

**Table S1.** sgRNA sequence and corresponding PAM sequence of MUC2, KAT6A and LMP1

| plasmid | SgRNA (5’-3’) | PAM (5’-3’) | Targeted gene |
| --- | --- | --- | --- |
| MUC2P | GGGGCACCTAGAGTGACCAG | AGG | MUC2 |
| KAT6A -sg1 | GGGCGCCTACCAGGACTGTG | AGG | KAT6A |
| LMP-g4 | GACCCGCCTTCGATGACAGA | CGG | LMP1 |

**Table S2 in the ESM**

**Table S2. The primer sequences of MUC2, LMP1, KAT6A and ODN**

| gene | forward primers | reverse primers |
| --- | --- | --- |
| MUC2 | CAGCACGTCATCCTGAAGGT | AAGACACCCTGGAGACACCT |
| KAT6A | CACGACGCTGATGATGAGGA | CACCTGCTGAGAGTGGTCTG |
| LMP1 | ACTCTGCTCTCAAAACCTAGGC | CATGTCCTCCTTTCCCCTTGTT |
| ODN | TTGAGTTGTCATATGTTAATAACGGT | ACCGTTATTAACATATGACAACTCAA |

**Table S3 in the ESM**

**Table S3.** The Top10 enriched GO terms of upregulated genes in PEI and PBAE groups.

| Description | P value |
| --- | --- |
| oxidoreduction-driven active transmembrane transporter activity | 0.000321 |
| MAP kinase tyrosine/serine/threonine phosphatase activity | 0.000594 |
| C3HC4-type RING finger domain binding | 0.000656 |
| calcium-dependent phospholipid binding | 0.000684 |
| clathrin binding | 0.001695 |
| protein tyrosine/threonine phosphatase activity | 0.002212 |
| MAP kinase tyrosine phosphatase activity | 0.002212 |
| RNA polymerase II activity | 0.002413 |
| ubiquinol-cytochrome-c reductase activity | 0.002676 |
| oxidoreductase activity, acting on a sulfur group of donors | 0.002686 |

***In vitro* hemolysis assay**

The red blood cells were obtained from the fresh blood of healthy C57 mice, and then were diluted with saline to prepare 2% red blood cell suspension. The 2% red blood cell suspension (250 μL) mixed with polymers-plasmid NPs solution (30 μL) and saline (220 μL) as test groups, and only mixed with saline (250 μL) as a negative control, and only mixed with water (250 μL) as the positive control. The above solutions of each group, set up 4 parallel samples, were incubated in a 37 ºC shaker for 2 hours, then were centrifuged (2000rpm,5min), and the supernatant was seeded into a 96-well plate, and detected on a microplate reader (λ=545 nm) to obtain the absorbance data. Hemolysis rate (%) = (absorbance of test solution group - absorbance of negative control group) / (absorbance of positive control group - absorbance of negative control group) × 100%.

**Preliminary evaluation of polymers-plasmids NPs in vivo exposure safety**

Preliminary evaluation safety of polymers-plasmids NPs in vivo exposure as follow：different concentrations of GSH-PBAE-plasmid, PBAE-plasmid and PEI-plasmid NPs were prepared，then the mice for each group were injected polymer-plasmid NPs (150 μl) into tail veins, and one mouse for one concentration of NPs. Then, the treated mice were observed for survival in 30 minutes.
